# TIRR regulates mRNA export and association with P-bodies in response to DNA damage

**DOI:** 10.1093/nar/gkae688

**Published:** 2024-08-09

**Authors:** Michelle S Glossop, Irina Chelysheva, Ruth F Ketley, Adele Alagia, Monika Gullerova

**Affiliations:** Sir William Dunn School of Pathology, Medical Sciences Division, University of Oxford, South Parks Road, Oxford OX1 3RE, UK; Oxford Vaccine Group, Department of Paediatrics, University of Oxford, and the NIHR Oxford Biomedical Research Centre, Oxford OX3 7LE, UK; Sir William Dunn School of Pathology, Medical Sciences Division, University of Oxford, South Parks Road, Oxford OX1 3RE, UK; Sir William Dunn School of Pathology, Medical Sciences Division, University of Oxford, South Parks Road, Oxford OX1 3RE, UK; Sir William Dunn School of Pathology, Medical Sciences Division, University of Oxford, South Parks Road, Oxford OX1 3RE, UK

## Abstract

To ensure the integrity of our genetic code, a coordinated network of signalling and repair proteins, known as the DNA damage response (DDR), detects and repairs DNA insults, the most toxic being double-strand breaks (DSBs). Tudor interacting repair regulator (TIRR) is a key factor in DSB repair, acting through its interaction with p53 binding protein 1 (53BP1). TIRR is also an RNA binding protein, yet its role in RNA regulation during the DDR remains elusive. Here, we show that TIRR selectively binds to a subset of messenger RNAs (mRNAs) in response to DNA damage. Upon DNA damage, TIRR interacts with the nuclear export protein Exportin-1 through a nuclear export signal. Furthermore, TIRR plays a crucial role in the modulation of RNA processing bodies (PBs). TIRR itself and TIRR-bound RNA co-localize with PBs, and TIRR depletion results in nuclear RNA retention and impaired PB formation. We also suggest a potential link between TIRR-regulated RNA export and efficient DDR. This work reveals intricate involvement of TIRR in orchestrating mRNA nuclear export and storage within PBs, emphasizing its significance in the regulation of RNA-mediated DDR.

## Graphical Abstract

**Graphical Abstract:**
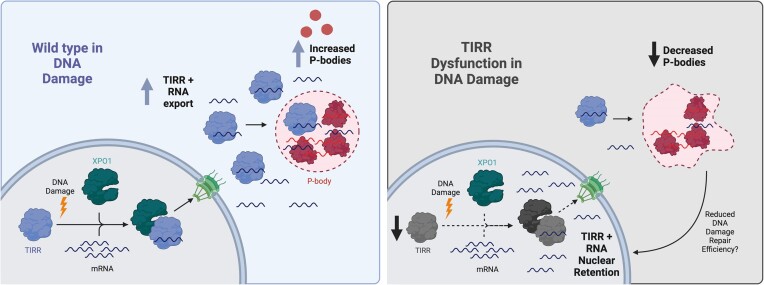

## Introduction

A large network of signalling and repair proteins, known as the DNA damage response (DDR), acts to ensure efficient repair caused by various damaging insults, including those resulting in DNA double-strand breaks (DSBs). Among these proteins, Tudor interacting repair regulator (TIRR), a NUDIX family protein (1), sits at the interface between homologous recombination (HR) and non-homologous end joining (NHEJ), two key pathways in DSB repair (2). TIRR binds and inhibits p53 binding protein 1 (53BP1), an integral factor in promoting NHEJ (3,4). 53BP1 counteracts resection, a step that is required to initiate HR, and is therefore extensively regulated. The role of TIRR in 53BP1 binding and regulation in the DSB repair response has been well characterized (5–9). In non-damage conditions, TIRR and 53BP1 form a complex. In response to DSBs, TIRR and 53BP1 dissociate, allowing 53BP1 to bind chromatin at the break (5). However, the wider role of TIRR outside of 53BP1 binding has not been extensively explored, especially in the context of DNA damage.

TIRR has been characterized as an RNA binding protein (6–9), binding a wide range of RNAs, particularly messenger RNA (mRNA) (6). Initial characterization of the role of TIRR in RNA binding in non-damage conditions suggests that TIRR can act as a translational repressor of the RNA to which it binds (6). Interestingly, TIRR was also identified as a precursor microRNA (pre-miRNA) binding protein in *in vitro* capture and mass spectrometry experiments, binding multiple different pre-miRNAs (7). Additionally, TIRR RNA binding is relevant in the context of DNA damage. We have previously shown that hairpin-shaped RNA derived from the DSBs contributes to the dissociation of TIRR from 53BP1 after DSB induction (8). Therefore, TIRR binds to a broad spectrum of RNA with distinct functional outcomes, yet this has been studied limitedly in the context of DNA damage.

RNA regulation and processing, including RNA transcription, stability, translation, storage and degradation, can be defined with the general term of RNA metabolism (10). RNA metabolism is extensively regulated in DDR, including global transcription inhibition of mRNA, alterations in splicing and degradation of transcripts, and production of damage-specific RNA species (11–15). Many different proteins act to regulate RNA metabolism, carefully coordinating RNA fate in different cellular contexts. For example, NUDIX family proteins participate in RNA metabolism (1), one of them being NUDT16, a close paralogue of TIRR involved in RNA de-capping (16). The regulation of RNA fate, such as decay and storage, can occur in different cellular compartments, including processing bodies (P-bodies or PBs). PBs are cytoplasmic membrane-less compartments that contain RNAs and various RNA binding proteins. In general, PBs are considered to be sites of non-translating mRNA (17,18) and sites of RNA decay or storage (17–19), promoting cell survival during and after stress (19,20). PBs contain components of the mRNA degradation machinery, such as DCP1/2, XNR1 and the CCR4–NOT complex, which are involved in mRNA de-capping and de-adenylation, as well as components of the miRNA pathway, such as GW182 and miRNAs (21). PBs can also associate with polysomes (22) and stress granules (SGs) (23), highly dynamic sites of mRNA storage and concentrated translation initiation factors. mRNAs can move between polysomes, where they are translated, and PBs, where they can be either stored or degraded, and SGs where they may be reintroduced to translation initiation factors, in a model known as the mRNA cycle (22,20,24).

Given that TIRR is an RNA binding protein involved in DSB repair, and DNA damage is known to impact RNA metabolism/functions, we wanted to assess whether TIRR may play a role in the regulation of RNA metabolism in response to DNA damage, as little is known about the function of TIRR in RNA regulation. TIRR could potentially participate at any stage of RNA metabolism, such as transcription, splicing and export from the nucleus, translation into proteins and RNA decay. To investigate this, we first performed RNA immunoprecipitation and sequencing (RIP-seq) experiments to identify specific RNAs bound to TIRR after DNA damage. Our analysis revealed that TIRR selectively binds to a subset of mRNAs. Furthermore, we demonstrate that a fraction of TIRR is exported from the nucleus through Exportin-1 (XPO1) binding upon DNA damage. TIRR localizes to P-bodies with its bound mRNAs and participates in the regulation of their formation in response DNA damage.

In essence, TIRR emerges as a pivotal player orchestrating the intricate features of mRNA regulation in the wake of genomic insults.

## Materials and methods

### Cell culture

Flp-IN TIRR-GFP, GFP, shTIRR and shGFP cells, wild-type (WT) HeLa cells, lentivirus-integrated TIRR-GFP WT, RNA binding mutant (RBM), 53BP1 binding mutant (K10E), NES1, NES2, NES3, NES4 and WT U2OS cells were cultured in Dulbecco’s modified Eagle medium containing 1% l-glutamine and 10% fetal bovine serum (FBS) at 37°C with 5% CO_2_ with and without antibiotics. TIRR-GFP, GFP, shTIRR and shGFP cells were a gift from Rosario Avolio. Drugs were added to cell culture media as indicated: doxycycline (1 μg/ml, 16–48 h) and leptomycin B (LMB; 5 nM, 24 h). Etoposide (ETO) (5–10 μM, 2 h) was used to induce DSBs. Etoposide at 5 μM concentration was used for highly sensitive RNA sequencing (RNA-seq) experiments to reduce non-specific perturbations in RNA expression and 10 μM was used for further, less sensitive experiments. DSBs were also induced using ionizing radiation (IR) at 10 Gy using a Gravatom (Gravitron RX 30/55), followed by incubation at 37°C for the specified times.

### Generating lentivirus-integrated cell lines

Lentivirus transfer plasmids were generated for GFP only, and TIRR-GFP WT, RBM, 53BP1 binding mutant (K10E), NES1, NES2, NES3 and NES4 using the lentivirus transfer plasmid backbone derived from pHR-mCh-Cry2WT. pHR-mCh-Cry2WT was a gift from Clifford Brangwynne (Addgene plasmid # 101221; http://n2t.net/addgene:101221; RRID:Addgene 101221). HEK293-T cells at ∼70% confluency in antibiotic-free media were co-transfected with one of the GFP/TIRR-GFP lentivirus transfer plasmid constructs, pMDG (envelope) and p8.91 (packaging) 25, using Lipofectamine 3000 (Thermo). p8.91 and pMDG were a gift from Simon Davis (Addgene plasmid # 187441; http://n2t.net/addgene:187441; RRID:Addgene_187441 and Addgene plasmid # 187440; http://n2t.net/addgene:187440 RRID:Addgene_187440) (25). After 48 h, packaging medium was harvested, and cellular debris was removed with a 0.45-μm filter. Packaging medium was added to U2OS cells at ∼70% confluency for 24 h. GFP-positive cells were enriched through FACS with a BD FACSAria III. Cell lines were maintained as described earlier.

### Cloning and plasmids

All cloning was performed using Gibson cloning with the NEBuilder^®^ HiFi DNA Assembly Master Mix (New England Biolabs). The PAGFP-TIRR plasmid was generated by amplifying a PAGFP fragment from a pPAGFP-C1 plasmid and a backbone containing TIRR using Q5^®^ High-Fidelity DNA Polymerase and associated Q5^®^ Reaction Buffer and GC Enhancer (New England Biolabs). The PAGFP fragment was inserted C-terminally to TIRR.

pPAGFP-C1 was a gift from Jennifer Lippincott-Schwartz (Addgene plasmid # 11910; http://n2t.net/addgene:11910 RRID:Addgene_11910) (26). The LSM14A-mScarlet plasmid was generated similarly, inserting mScarlet from an existing TIRR-mScarlet plasmid N-terminally into an LSM14A-containing backbone derived from an LSM14A-GFP plasmid. The LSM14A-GFP plasmid was a gift from the Weil Lab at Sorbonne Université (24).

Due to the GC-rich sequence of TIRR, the RBM and NES (nuclear export signal) mutants were generated by another method. For the RBM, forward and reverse 180-nt single-stranded DNA (ssDNA) fragments with overlapping 3′ complementary regions were designed to contain individually mutated residues according to (27). For the NES mutants, each ssDNA fragment contained either a WT TIRR sequence or a mutated NES site, in which all hydrophobic residues were mutated to alanine (alanine scanning) and the surrounding TIRR sequence. All ssDNA fragments were produced as Ultramers™ from IDT. These ssDNA fragments were then annealed and extended to form a double-stranded DNA fragment containing either RBM TIRR or mutated NES sites that were sufficiently large for efficient Gibson cloning. These fragments were amplified along with a GFP-containing plasmid backbone. Cloning was performed as described earlier. All constructs were verified with Sanger sequencing (Source BioScience). mCherry-LaminB1-10 was a gift from Michael Davidson (Addgene plasmid # 55069; http://n2t.net/addgene:55069; RRID:Addgene 55069). Flag-hCRM1 (FLAG-XPO1) was a gift from Xin Wang (Addgene plasmid # 17647; http://n2t.net/addgene:17647; RRID:Addgene 17647) (28). The plasmid mScarlet-FLAG was created by Adele Alagia using mScarlet from PH-PLCD1_mScarlet-I_IRES_sYFP2_PH_N1, a gift from Dorus Gadella (Addgene plasmid # 110623; http://n2t.net/addgene:110623; RRID:Addgene 110623).

For oligonucleotide sequences, see Supplementary Table S3.

### Western blot

Western blotting was performed as in (8). Protein samples were boiled in Laemmli buffer for 7 min at 98°C, run on 4–15% Mini-Protean TGX gels (Bio-Rad) at 100 V for ∼1.5 h and transferred onto nitrocellulose membranes using a Trans-Blot Turbo Transfer System (Bio-Rad). Membranes were blocked in 5% milk in PBST (Triton X-100 in phosphate-buffered saline) for 1 h, before incubation with primary antibodies overnight at 4°C. Secondary antibody was incubated for 1 h at room temperature (RT). Membranes were visualized using ECL (Thermo) and developed on an Amersham Hyperfilm ECL film. Primary antibodies used were as follows: GFP Monoclonal antibody (3H9, Chromotek) 1:8000–10 000, Rb pAb to beta-tubulin (Abcam) 1:2000–4000, GAPDH Monoclonal antibody (Proteintech) 1:2000–4000, Rabbit anti-53BP1 pAb (Novus Biologicals) 1:500, Anti-phospho-Histone H2A.X (Ser139) Antibody clone JBW301 (Millipore) 1:1000, Anti-NUDT16L1 (Atlas Antibodies) 1:500, Anti-LSM14A Purified MaxPab antibody (Novus Biologicals) 1:250, CRM1 C-1 antibody (Santa Cruz) 1:250, mCherry antibody 1:1000 (GeneTex), Rb pAb to LaminB1 (Abcam) 1:1000, Anti-FLAG antibody produced in Rabbit (Sigma) 1:1000 and Histone-H3 Polyclonal Antibody (Proteintech) 1:8000–1:10 000. NUDT16L1 antibody (Atlas Antibodies) and CRM1 antibody (Santa Cruz) were also incubated with SuperSignal™ Western Blot Enhancer (Thermo Fisher).

### RNA immunoprecipitation and sequencing

3 × 15-cm dishes were used per condition and plated to achieve ∼70% confluency at the point of harvest. TIRR-GFP and GFP cells were plated and 24 h later induced with 1 μg/ml doxycycline overnight to induce expression. Cells were washed three times in cold PBS and cross-linked at 254 nm at 0.15 J/cm^2^. Cells were lysed in cold RIP lysis buffer (100 mM NaCl, 15 mM MgCl_2_, 10 mM Tris, pH 7.5, 0.5% NP-40, 0.5% sodium deoxycholate, 1 mM dithiothreitol) with 1× protease inhibitor (Roche) and RiboLock (Thermo), with Turbo DNase (Invitrogen). Lysates were sonicated (10 s on, 30 s off) at medium intensity. Lysates were centrifuged at 17 000 × *g* for 10 min and the supernatant collected. GFP-Trap A beads were washed three times in lysis buffer and added to samples for 2 h on a wheel at 4°C. Beads were collected by centrifugation at 2500 × *g* for 2 min and washed two times with RT RIP high-salt buffer (500 mM NaCl, 1 mM MgCl_2_, 20 mM Tris, pH 7.5, 0.05% NP-40, 0.1% sodium dodecyl sulfate, protease inhibitor), two times with cold RIP low-salt buffer (150 mM NaCl, 20 mM Tris, pH 7.5, 1 mM MgCl_2_, 0.01% NP-40) and two times with cold RIP Proteinase K buffer (100 mM Tris, pH 7.5, 50 mM NaCl, 10 mM ethylenediaminetetraacetic acid (EDTA)]. Beads were incubated with Proteinase K (Thermo) in RIP Proteinase K buffer at 37°C for 1 h with shaking at 1100 rpm. RNA was extracted using TRIzol and the Monarch Total RNA Miniprep Kit according to the manufacturer’s instructions. RNA samples were submitted to the Oxford Genomics Centre for ribosomal RNA (rRNA) depletion and paired-end bulk full transcript RNA-seq on the Illumina NovaSeq6000 was performed.

### Total RNA extraction

Total RNA was extracted by scraping from plates using RNA lysis reagent from the Monarch Total RNA Miniprep Kit. RNA was eluted in water and quantified using an Implen NanoPhotometer N60.

### RNA sequencing

Total RNA-seq was performed on stably integrated inducible short hairpin RNA (shRNA) HeLa cells, expressing shRNA for TIRR or GFP. TIRR knockdown (KD) was induced using doxycycline for 48 h. RNA was collected before and after DNA damage, induced by IR and followed by 1 h incubation, or by ETO incubation for 2 h. It was then subjected to paired-end total RNA-seq in three replicates at Azenta Life Sciences on the Illumina NovaSeq6000.

### Fluorescence *in situ* hybridization/immunofluorescence

TRex Flp-IN shTIRR and TRex Flp-IN shGFP HeLa cells were plated on poly-l-lysine-coated glass coverslips. TIRR KD was obtained by a doxycycline-inducible shRNA against TIRR (6). Coverslips were then washed three times in cold PBS and fixed in cold 4% paraformaldehyde (Alfa Aesar) in PBS for 15 min. Coverslips were then washed three times in cold PBS and permeabilized with cold 0.2% PBST for 10 min. Slides were then washed three times in cold PBS and blocked in fluorescence *in situ* hybridization (FISH) blocking buffer [2× SSC, 40 ng/μl yeast transfer RNA (tRNA; Life Technologies), 40 ng/μl salmon sperm DNA (Life Technologies), 0.5% Triton X-100, 2% bovine serum albumin (BSA) and 0.7 U/μl RNasin Plus (Promega)] at 37°C for 1 h. Coverslips were incubated with FISH probe incubation buffer [2× SSC, 40 ng/μl yeast tRNA (Life Technologies), 40 ng/μl salmon sperm DNA (Life Technologies), 0.5% Triton X-100, 2% BSA, 1 U/μl RNasin Plus (Promega) and 100 nM DNA probes] for 3 min at 95°C, and then at 4°C overnight in a humidified chamber. Coverslips were washed three times for 10 min at RT in 2× SSC-T buffer (2× SSC, 0.05% Triton X-100), three times in 1× SSC-T buffer (1× SSC, 0.05% Triton X-100), three times in 0.5× SSC-T buffer (0.5× SSC, 0.05% Triton X-100) and three times in 0.25× SSC-T buffer (0.25× SSC, 0.05% Triton X-100). Coverslips were then incubated with immunofluorescence (IF) blocking buffer (2× SSC, 5% FBS, BSA 5BSA) for 1 h at 37°C. LSM14A primary antibody (18336-1-AP, Proteintech) was diluted to the appropriate concentration in 5% FBS and 5% BSA in 2× SSC and incubated overnight at 4°C in a humidified chamber. Coverslips were then washed three times in SSC-T buffer (2× SSC, 0.1% Triton X-100) and subsequently incubated for 2 h at RT with Donkey anti-Rabbit Alexa Fluor™ 555 (A32794, Invitrogen) secondary antibodies diluted in 0.1% FBS and 0.1% BSA in 2× SSC. Coverslips were washed three times in 2× SSC and 0.1% Triton X-100 and once in 2× SSC before mounting on slides with Fluoroshield Mounting Medium with DAPI (Abcam). After imaging, brightness and contrast were increased equally across images to enhance visibility of foci against the background. Overlap between FISH signal foci and LSM14A IF foci was called, identifying notably yellow foci and excluding faint foci. The overlapped foci were quantified as a percentage of total P-body number.

### Confocal microscopy

IF and confocal microscopy were performed according to a previously published protocol (8). Cells were plated on glass coverslips and fixed in cold 4% paraformaldehyde in PBS for 10 min. Cells were permeabilized with cold 0.2% Triton X-100 in PBS for 10 min, followed by blocking in 10% FBS in PBS for 2 h at 4°C. Primary antibodies were diluted in 10% FBS in PBS and incubated overnight at 4°C in a humidified chamber. Secondary antibody incubation was performed for 2 h at RT with secondary antibodies diluted in 0.15% FBS in PBS. Fluoroshield Mounting Medium with DAPI was used to mount slides on coverslips, and imaging performed using an Olympus Fluoview FV1200 confocal microscope with a 60× objective lens.

### Photoactivation and live cell microscopy

Live cell imaging was performed with a SoRa spinning disc confocal microscope. U2OS cells were reverse transfected with 1 μg each of PAGFP-TIRR and mCherry-LaminB1-10 or LSM14A-mCherry plasmids of Lipofectamine LTX with PLUS reagent (Thermo Fisher) in plastic six-well plates and then reseeded on poly-l-lysine-coated glass-bottom 35-mm plates. Cells were treated with LMB where stated, and damage was induced the following day with IR. Plates were immediately brought to the microscope for imaging. PAGFP-TIRR was induced by 405 nm laser, which was followed by time-lapse imaging, using 488 and 555 nm lasers for PAGFP-TIRR and mCherry-LaminB1-10, respectively. Images were taken every 4 s for 2 min. Quantification of the cytoplasmic signal was performed using FIJI software.

### Proximity ligation assay

Proximity ligation assay (PLA) was performed according to the manufacturer’s instructions and our previously published protocol (8).

### Fluorescence *in situ* hybridization and proximity ligation assay

GFP, TIRR-GFP WT or TIRR-GFP mutant lentivirus-integrated U2OS cells were seeded at ∼60% confluency on glass coverslips and treated as described. The specifics of the FISH–PLA (fluorescence *in situ* hybridization and proximity ligation assay) technique used in this manuscript were adapted from (29). Cells were fixed in 4% paraformaldehyde in PBS at 37°C for 10 min. Cells were permeabilized with cold 0.5% Triton X-100 in PBS for 10 min at RT. Blocking was performed using an RNAse-free FISH–PLA blocking buffer, including 2× SSC (Lifetech), 0.5% Triton (Sigma), 2% BSA (Sigma), 40 ng/μl salmon sperm DNA (Life Technologies) and 0.4 U/μl RNasin Plus (Promega) at 37°C for 1 h in a humidity chamber. Five to six probes per mRNA target were diluted to 30 nM in 2× SSC and heated at 98°C for 3 min followed by snap cooling on ice. Probes were designed with 40–50 nt of highly specific homology to the transcript of interest, followed by a linker region and a region corresponding to the PLUS probe found in the Duolink^®^ Proximity Ligation Assay Kit (Sigma–Aldrich). 0.5% Triton, 0.8 U/μl RNasin Plus and 20 ng/μl SSSDNA were added. Coverslips with cells were incubated with probes for 3 min at 95°C, and then 2.5 h at 37°C on glass slides in a humidity chamber. Cells were then washed three times with RT 2× SSC buffer (2× SSC, 2% Triton, 1% BSA), three times with 1× SSC buffer (1× SSC, 2% Triton, 1% BSA) and once with PBS. PLA was then performed according to the manufacturer’s instructions and our previously published protocol (8), beginning with blocking with the provided blocking buffer. Cells were incubated with only one antibody [Anti-GFP antibody (Abcam) 1:500] and only the anti-rabbit MINUS secondary antibody in the PLA kit.

### γ-H2AX IF time course

Approximately 1 000 000 cells each of U2OS stably expressing GFP, TIRR-GFP WT, TIRR-GFP K10E and TIRR-GFP NES2 were reverse transfected with 20 nM small interfering RNA (siRNA) against the 3′ untranslated region (UTR) of endogenous TIRR (Dharmacon, ON-TARGETplus Human NUDT16L1 siRNA, J-014872-23-0005) using 3 μl of Lipofectamine RNAiMAX (Thermo Fisher) in a six-well plate. Twenty-four hours later, the cells were split into six-well plates containing poly-lysine coating. Twenty-four hours later, cells were irradiated with 10 Gy. Samples were collected at 15 min and 6 h and processed according to the methods described in the ‘Confocal microscopy’ section.

### Co-immunoprecipitation

Co-immunoprecipitation (co-IP) was performed according to a previously published method (8). For NES mutant analysis, stably integrated lentivirus GFP, TIRR-GFP WT and mutant cell lines were seeded according to expression levels of each construct (adjusted from ∼4 million for WT) and treated as described. Briefly, cells were lysed in lysis buffer [50 mM Tris, pH 7.5, 150 mM NaCl, 1 mM EDTA, 5 mM MgCl_2_, 0.5% NP-40, protease and phosphatase inhibitors (Roche), RiboLock RNase Inhibitor (Thermo) and Turbo Nuclease (Thermo)] on a wheel at 4°C for 20 min, 7 min at RT and then on a wheel at 4°C for 10 min, before centrifugation at 17 000 × *g* for 10 min. Lysates were diluted in lysis buffer without NP-40 and Turbo Nuclease (1.5× cell lysate volume) and incubated with GFP-Trap magnetic beads (Chromotek) for 1.5 h at 4°C. Beads were washed in lysis buffer without NP-40 and Turbo Nuclease and samples were eluted using 2× Laemmli buffer and boiling at 95°C. For XPO1-Flag co-IP, 2 million U2OS cells were first transfected with 1 μg of plasmid using LTX and PLUS reagent (Thermo Fisher). Twenty-four hours later, cells were transferred to 10-cm dishes. Sixteen hours later, cells were collected and co-IP was performed as described earlier using Anti-FLAG^®^ M2 magnetic beads.

### *In vitro* binding assay of TIRR and XPO1

Ni-NTA magnetic agarose beads were incubated with 1.5 μg of recombinant His-TIRR (8) in binding buffer [50 mM Tris, pH 7.4, 150 mM NaCl, 5 mM MgCl_2_, 10% glycerol, protease inhibitors (EDTA-free, Roche)] for 1 h at 4°C on a wheel. Beads were washed two times in binding buffer and incubated with 6 μg recombinant GST-tagged XPO1 (ab131897, Abcam) for 1 h at 4°C on a wheel. The supernatant was removed and the beads were washed four times in binding buffer. Proteins were eluted in 500 mM imidazole and eluant was collected and ran on a western blot to visualize binding.

Details about oligonucleotides used in this study can be found in Supplementary Table S3.

### Data and statistical analysis

#### RNA-seq analysis

The sequencing data were aligned against the whole human (*Homo sapiens*) genome build GRCh38 (https://ccb.jhu.edu/software/hisat2/index.shtml), using STAR (version 2.7.3a). Gene features were counted using HTSeq (version 0.11.1), using human gene annotation general transfer format version GRCh38.92 (www.ensembl.org). Genes with low counts across most libraries were removed. rRNA, sex chromosome genes, mitochondrial RNA and haemoglobin genes were excluded from downstream analysis. Differential gene expression was conducted using the R Bioconductor packages ‘edgeR’ and ‘limma’ (30–34). RNA-seq data were then normalized for RNA composition using the trimmed mean of *M*-value method (31). Data were transformed using the limma ‘voom’ function. A linear model was fitted to the data using the limma ‘lmFit’ function using the empirical Bayes method (32). The cut-off for statistical significance was set at a false discovery rate (FDR) of <0.05. Principal component analysis (PCA) and heatmaps were performed using the R Bioconductor package ‘pcaExplorer’ (33) on the log transformed data; confidence interval level of 0.95 has been used. Gene Ontology (GO)-term enrichment analysis and characterization of the gene lists compared to the whole genome were performed in ShinyGO (version 0.76) (35) using GO Biological process and GO Molecular function databases, where specified. The cut-off for statistical significance was set at an FDR of <0.05; up to 20 top pathways were shown with redundant pathways removed.

#### Statistical tests

To test for a normal distribution, the Kolmogorov–Smirnov normality test was performed. If data met the requirements of a normal distribution, an unpaired *t*-test was performed. For comparison of two groups if data did not show a normal distribution, a Mann–Whitney test was performed. For comparison of more than two groups, a Kruskal–Wallis with Dunn’s multiple comparisons test was carried out. *P*> 0.05 (ns), *P ≤ 0.05, **P ≤ 0.01, ***P ≤ 0.001 and ****P ≤ 0.0001. GraphPad Prism 9 was used to perform the statistical analysis.

## Results

### TIRR binds to specific transcripts in response to DNA damage

**Figure 1.**
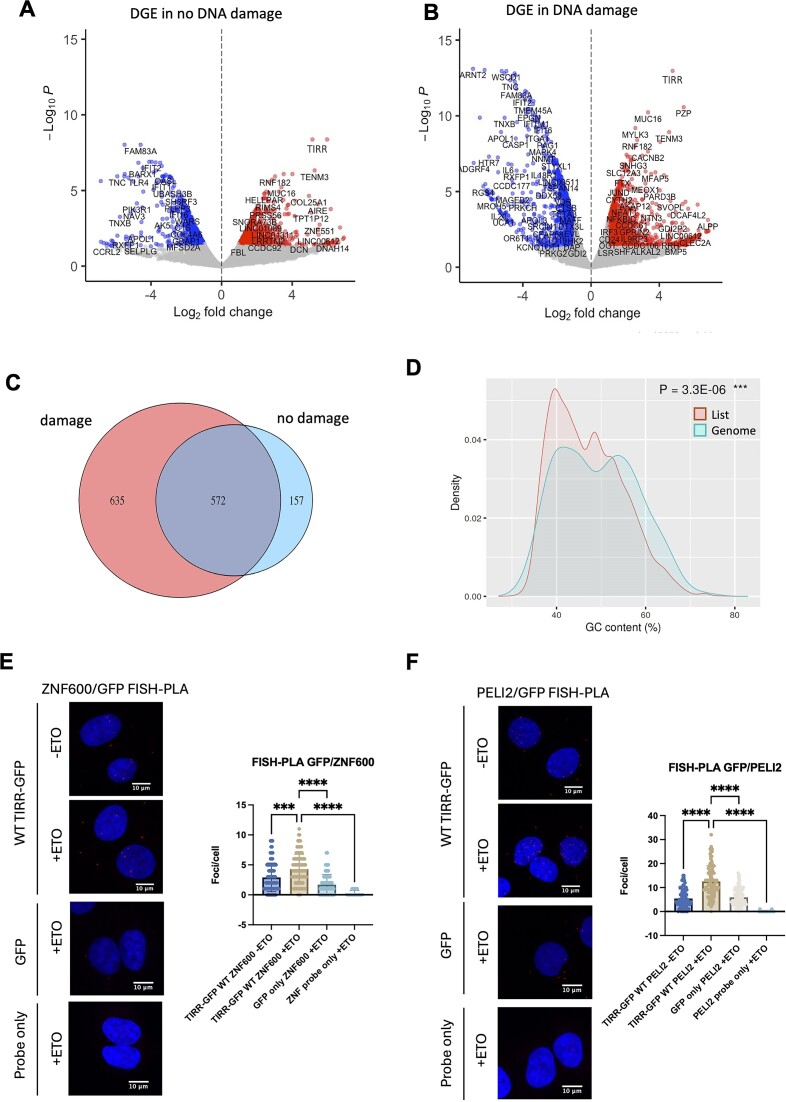
TIRR binds to mRNA of transcription-associated proteins upon DNA damage. (**A**) Volcano plot showing differentially enriched RNAs in TIRR-GFP IP over GFP-only IP in non-damage conditions: TIRR-GFP IP (*n* = 2) and GFP IP (*n* = 1). (**B**) As in panel (A), in DNA damage conditions. (**C**) Pie chart showing an overlap between RNA transcripts identified by TIRR RIP-seq in non-damage conditions and DNA damage conditions. (**D**) Comparison of GC content feature of RNAs bound to TIRR in damage conditions versus the reference genome. (**E**) Left: FISH–PLA of *ZNF600* with GFP in a WT TIRR-GFP stable cell line +/− ETO damage and a GFP-only stable cell line + ETO, and a *ZNF600* probe-only control + ETO. Right: Quantification, *n* > 80 cells. Significance was determined using the Kruskal–Wallis test (***P ≤ 0.001, ****P ≤ 0.0001). (**F**) As in panel (E), for *PELI2*.

To explore how TIRR may function as an RNA binding protein in DNA damage, we first performed RNA immunoprecipitation with ultraviolet (UV) cross-linking followed by sequencing, allowing us to identify specific RNA bound to TIRR in DNA damage conditions (36–38) (Supplementary Figure S1A). UV light at 254 nm directly cross-links RNA and protein, but not protein and protein, allowing for stringent washing conditions during immunoprecipitation to remove non-specific RNA. To perform the RIP-seq, TIRR-GFP or GFP only (to control for background) was overexpressed through doxycycline induction in HeLa cells with integrated inducible TIRR-GFP or GFP-only constructs (referred to as TIRR-GFP OE and GFP OE cell lines) (Supplementary Figure S1B and C). This was followed by the introduction of DSBs using ETO, and subsequent RIP-seq (Supplementary Figure S1D–H). A relatively low dose of 5 μM ETO for 2 h was used to reduce the impact of any non-specific effects of drug treatment in the highly sensitive RNA-seq. PCA revealed good reproducibility of the RIP-seq samples, although there is some variability in the samples isolated from TIRR-GFP OE non-damage replicates (Supplementary Figure S1F). The variability observed in TIRR non-damage samples may reduce the number of significantly differently expressed genes for this condition compared to the GFP non-damage condition, and consequentially decrease the scope of damage-specific TIRR-GFP interacting transcripts identified through this RIP-seq. FISH–PLA was employed to validate the change in interaction of selected transcripts, *PELI2* and *ZNF600*, with TIRR between non-damage and DNA damage conditions. Instead of detecting the proximity of two proteins as in PLA, FISH–PLA detects proximity between a certain transcript and a certain protein, using probes specific to RNA linked to a PLUS PLA probe sequence (Supplementary Figure S1I) (29). FISH–PLA was performed on U2OS cells stably expressing lentivirus-integrated GFP and TIRR-GFP WT in ETO-treated and non-treated conditions. In damage conditions, foci increased significantly from non-damage in TIRR-GFP WT and decreased dramatically in GFP only (Figure 1E and F). Probe-only controls also revealed a near-total loss of foci. Additional non-TIRR-bound control transcripts, *SPEN* and *Tubulin*, also revealed no increase in interaction upon DNA damage (Supplementary Figure S1J and K). Therefore, we confirm increased interaction between *ZNF600* and TIRR, and *PELI2* and TIRR upon DSB induction.

In total, we identified 729 RNAs bound to TIRR in non-damage conditions, and 1207 RNAs bound to TIRR after damage. Intersection of these RNAs revealed an overlap of 572 RNAs. Interestingly, we identified 635 RNAs uniquely bound to TIRR upon DNA damage (Figure 1A–C and Supplementary Table S1). GO-term analysis of TIRR-bound RNAs found in both damage and non-damage conditions showed an enrichment in biological processes, including mRNA processing and mRNA splicing, suggesting a potential role for TIRR in RNA metabolism (Supplementary Figure S2B). GO analysis of RNAs that were bound to TIRR specifically after damage showed an enrichment for pathways involved in the regulation of RNA polymerase II (RNAPII) transcription, including DNA binding transcription factor activity, sequence-specific DNA binding and RNAPII transcription regulatory region sequence-specific binding (Supplementary Figure S2A, C and D). This includes a subset of zinc finger-containing transcription factors (Supplementary Figure S2C and D). Further analysis of RNAs bound to TIRR after damage showed no significant difference in 5′ UTR length, transcript length or 3′ UTR length relative to the rest of the genome (Supplementary Figure S3). However, analysis of the GC content of RNAs bound to TIRR revealed a significant enrichment of mRNAs with lower GC content (Figure 1D), which could be relevant to their metabolism (39). It should be noted that, as the range of transcripts identified through this RIP-seq is reduced due to reproducibility issues, additional features of TIRR-bound transcripts may have been missed.

To assess what role TIRR may serve in binding to this subset of mRNAs corresponding to RNAPII regulatory factors, we first performed total RNA-seq in stably integrated doxycycline-inducible shRNA HeLa cells, expressing shRNA for TIRR (shTIRR) or GFP (shGFP) as a control (Supplementary Figure S4A and B). TIRR KD was induced using doxycycline for 48 h, before inducing damage by treatment with ETO (5 μM) for 2 h. Total RNA was collected and subjected to paired-end total RNA-seq. Heatmap analysis and PCA revealed similarity between the RNA-seq replicates (Supplementary Figure S4C and D). We observed that TIRR RNA was successfully depleted in our sequencing experiments due to a significant decrease in the relative expression of TIRR/NUDT16L1 RNA after TIRR KD (Supplementary Figure S4E). Globally, TIRR KD did not result in significant changes in total RNA levels, with very few RNAs showing log_2_FC > 1 or log_2_FC < −1 and FDR < 0.05, suggesting that TIRR does not have a large influence on steady-state RNA levels. Many of the same RNAs were similarly differentially expressed in TIRR KD in non-damage conditions compared to damage conditions (Supplementary Figure S4F–I and Supplementary Table S2), suggesting that TIRR KD does not dramatically impact steady-state RNA levels either generally or in a damage-specific manner. We also performed RNA-seq of samples in which damage was induced by IR (10 Gy) followed by a 1-h incubation period, to see whether a more temporally concentrated dose of DNA damage could yield different results; however, we observed a similar lack of a significant change in steady-state RNA levels (Supplementary Figure S5A–F). Comparison of both RNA-seq datasets (ETO and IR) revealed similarity in differentially expressed genes (DEGs) under these two conditions (Supplementary Figure S5G). Comparison of the RIP-seq data with our RNA-seq data showed that of the 1207 RNA transcripts bound in damage conditions, only 3 were found to be significantly differentially expressed in TIRR KD in ETO damage compared to non-damage conditions (>1%). Overall, we conclude that TIRR binding to RNA does not dramatically influence the total steady-state levels of RNA globally.

To further explore the impact TIRR binding may have on these transcripts, we also selected two proteins encoded by these transcripts to analyse by western blot in damage and TIRR KD conditions. HeLa cells expressing shTIRR or shGFP as a control were induced with doxycycline and treated with ETO to induce DNA damage or left untreated. In all conditions, protein levels did not change significantly (Supplementary Figure S6A–C), suggesting that TIRR does not directly regulate protein production of bound transcripts.

### TIRR depletion leads to mRNA retention in the nucleus

**Figure 2.**
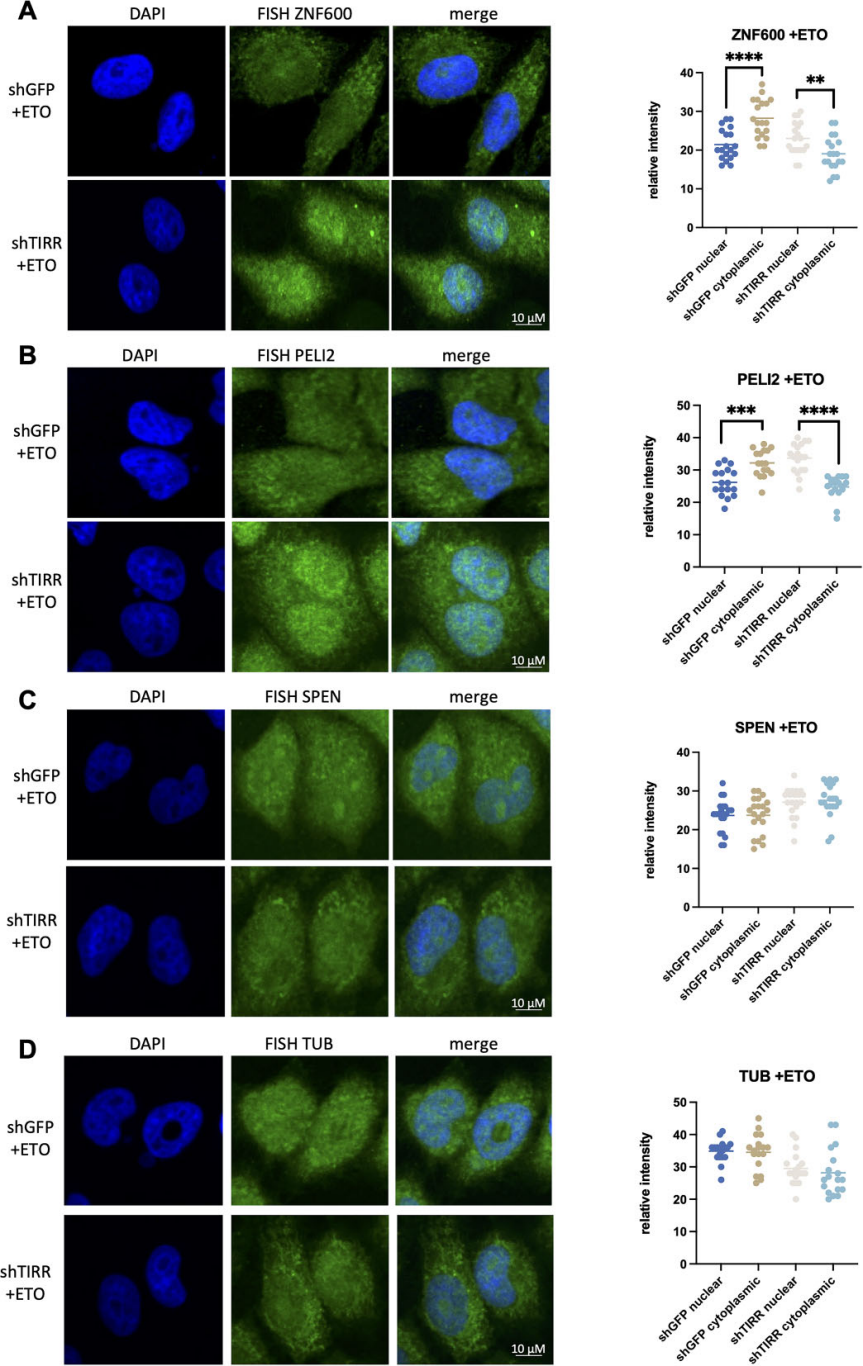
TIRR depletion leads to mRNA retention in the nucleus. (**A**) Left: Confocal images showing RNA FISH signals (middle panels, merged with DAPI in right panels) for *ZNF600* in shGFP or shTIRR cells after ETO treatment. Right: Quantification of *ZNF600* mRNA signals in nucleus and cytoplasm plotted as relative FISH signal intensity in shGFP or shTIRR cells upon ETO treatment (*n* > 50 cells). Significance was determined using a *t*-test (***P ≤ 0.001, ****P ≤ 0.0001). (**B**) As in panel (A), for *PELI2* mRNA. (**C**) As in panel (A), for *SPEN* mRNA. (**D**) As in panel (A), for *Tubulin* mRNA.

As TIRR did not appear to influence steady-state RNA levels and selected protein levels of mRNA bound to TIRR upon damage, we instead explored whether it may impact localization of bound mRNAs and potentially influence their fate. We performed FISH using DNA probes for selected mRNAs, *ZNF600* and *PELI2*, identified to be specifically bound to TIRR in damage conditions in our RIP-seq and validated by FISH–PLA. FISH was also performed on two negative controls, *SPEN* and *Tubulin*, both mRNAs not found to bind TIRR in damage. FISH was performed in shTIRR and shGFP cells in non-damage and ETO-induced damage conditions. While a relatively low dose of 5 μM ETO was used to reduce the impact of any non-specific effects of drug treatment in the highly sensitive RNA-seq, a more typical dose of 10 μM was used from here on (Supplementary Figure 6D and E). We observed that upon damage, both *ZNF600* and *PELI2* mRNAs were predominantly localized in the cytoplasm in shGFP control cells. However, depletion of TIRR resulted in significant *ZNF600* and *PELI2* mRNA nuclear retention (Figure 2A and B). Such a TIRR-dependent change in localization was not observed in FISH of our negative controls, *SPEN* or *Tubulin* (Figure 2C and D). In the non-damage conditions, nuclear retention was also observed upon TIRR KD; however, this was less pronounced than that in damage conditions (Supplementary Figure S7A–D). Similar to these observations, we also detected TIRR-dependent cytoplasmic localization of *ZNF600* and *PELI2* mRNAs upon IR treatment (Supplementary Figure S8A and B). These data suggest that TIRR might be required for nuclear export of specific mRNAs that it binds upon DNA damage induction.

### TIRR re-localizes from nucleus to cytoplasm upon DNA damage

**Figure 3.**
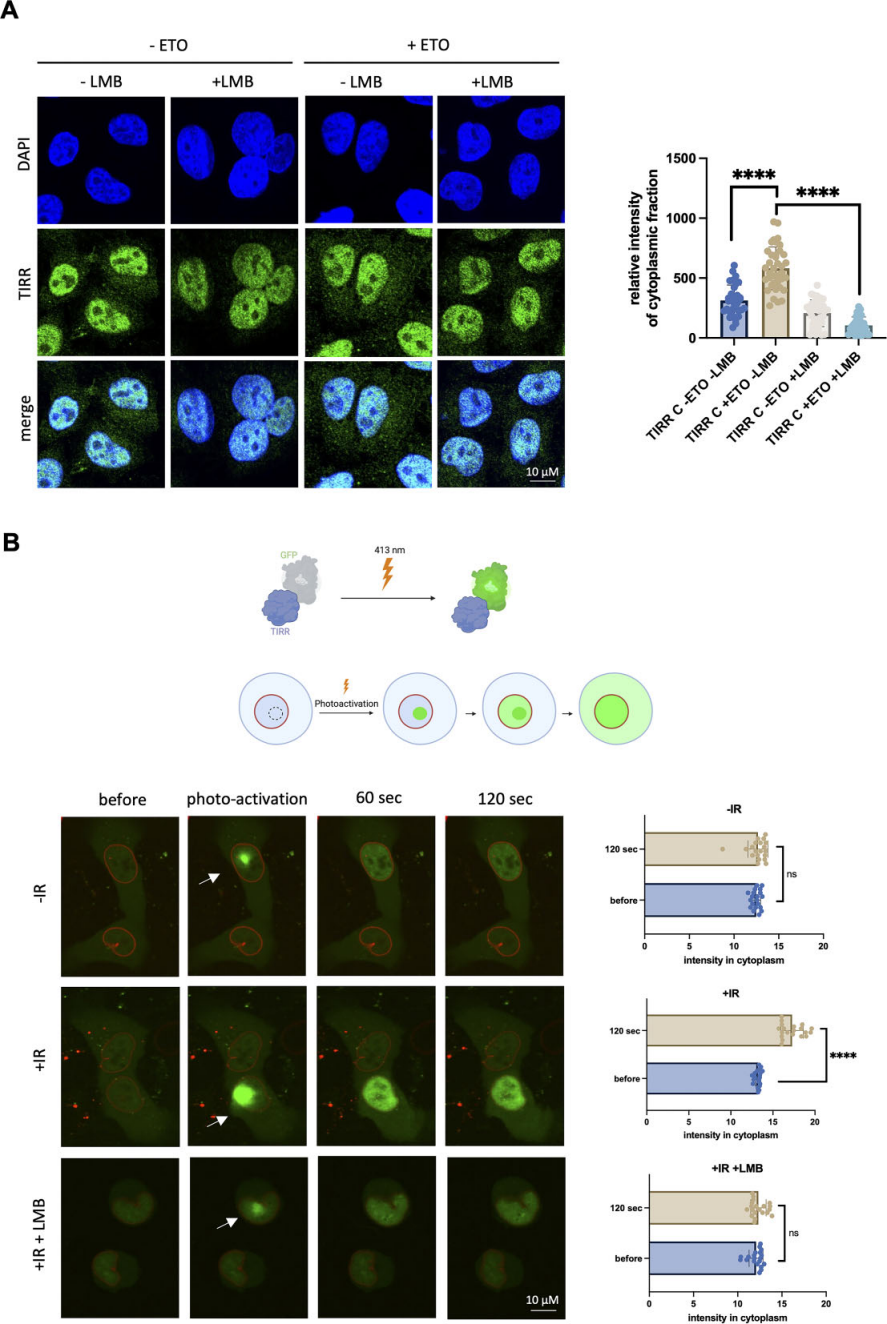
TIRR re-localizes from nucleus to cytoplasm upon DNA damage. (**A**) Left: Representative confocal images showing IF of TIRR in HeLa cells with or without ETO treatment and with or without LMB treatment. DAPI was used to stain nuclei. Right: Quantification of TIRR signal in cytoplasm (*n* > 50 cells); significance was determined using a *t*-test (****P≤ 0.0001). (**B**) Top: Diagram showing photoactivation strategy. Bottom: Representative confocal images showing live cells expressing PAGFP-TIRR (nuclear and cytoplasmic signal); mCherry-LaminB1-10 was used as a marker for the nuclear envelope (outlines nucleus). Photoactivation was induced using 413 nm laser and the location of activation is indicated with a white arrow. Photoactivation was followed by time-lapse microscopy, with time points at 60 and 120 s shown. Quantification of cytoplasmic GFP signal was performed using FIJI software. Significance was determined using a *t*-test (****P ≤ 0.0001). See also Supplementary Movies S1–S3. Illustration created with Biorender.com.

Having shown that selected damage-specific TIRR-bound transcripts require TIRR for cytoplasmic localization, we next examined whether TIRR itself may be exported to the cytoplasm upon DNA damage. TIRR exists in both the nuclear and cytoplasmic cellular compartments (5,6). Therefore, we examined whether nuclear export of TIRR may be regulated by the protein chromosome region maintenance protein 1, also known as XPO1/CRM1. XPO1 is the main protein export receptor in human cells (40), regulating the export of different proteins and some RNAs, including small nuclear RNA, tRNA and a subset of mRNAs, from the nucleus through the nuclear pore complex (NPC) into the cytoplasm (40,41). XPO1 associates with RAN-GTP to bind its cargo, exports through the NPC and, upon RAN-GTP hydrolysis to RAN-GDP, releases its cargo into the cytoplasm (41). XPO1-dependent RNA export is important for many cellular processes, such as translation and mRNA decay (42,43). To mediate RNA export, XPO1 does not bind directly to RNAs, but instead binds to proteins with a nuclear export signal (NES) that can themselves bind RNA, such as HuR (40,41), making XPO1 an interesting candidate for export of TIRR and its bound mRNA. In WT HeLa cells, we performed IF of TIRR in control and ETO-induced damage conditions in the presence and absence of LMB, which inhibits XPO1 activity by preventing its nuclear reimport (44). In the absence of LMB, we observed a significant increase in localization of TIRR to the cytoplasm upon DNA damage. Upon the addition of LMB, TIRR was retained in the nucleus, suggesting that TIRR export is mediated by XPO1 (Figure 3A). To validate our data further, we performed the same experiment in cells treated with IR to induce DNA damage and observed damage-dependent, LMB-sensitive, TIRR re-localization into the cytoplasm (Supplementary Figure S9A). To eliminate the possibility that this phenotype is cell type specific, we also performed this IF experiment in U2OS cells treated with ETO and detected damage-induced TIRR localization to the cytoplasm, which was inhibited by LMB treatment (Supplementary Figure S9B). To test the nuclear to cytoplasmic shift in real time, we employed a previously characterized engineered photoactivatable variant of GFP, which increases in fluorescence 100 times after irradiation with 413 nm light and allows for protein tracking *in vivo* (26). We generated a photoactivatable GFP-tagged TIRR construct (PAGFP-TIRR) and transfected U2OS cells with both PAGFP-TIRR plasmid and an mCherry-LaminB1-10 plasmid, chosen to delineate the borders of nuclei (Figure 3B, illustration, and Supplementary Figure S9C). To induce DNA damage, IR was chosen as it induces damage on a shorter, more concentrated timescale than ETO, allowing us to observe TIRR re-localization very quickly after damage induction. A combined IR damage induction and LMB treatment condition was also included. PAGFP was activated with 413 nm light at a single point within the nucleus followed by time-lapse imaging for 120 s. In non-damage conditions, GFP signal did not increase significantly in the cytoplasm over time (Figure 3B and Supplementary Movie S1). However, in damage conditions, GFP signal in the cytoplasm increased significantly (Figure 3B and Supplementary Movie S2). In the LMB-treated damage condition, no detectable increase in cytoplasmic GFP signal was observed (Figure 3B and Supplementary Movie S3), further confirming the importance of XPO1 in facilitating export even over a short timescale. These data suggest that DNA damage stimulates XPO1-mediated LMB-sensitive TIRR export from nucleus into cytoplasm.

### TIRR binds XPO1 upon DNA damage

**Figure 4.**
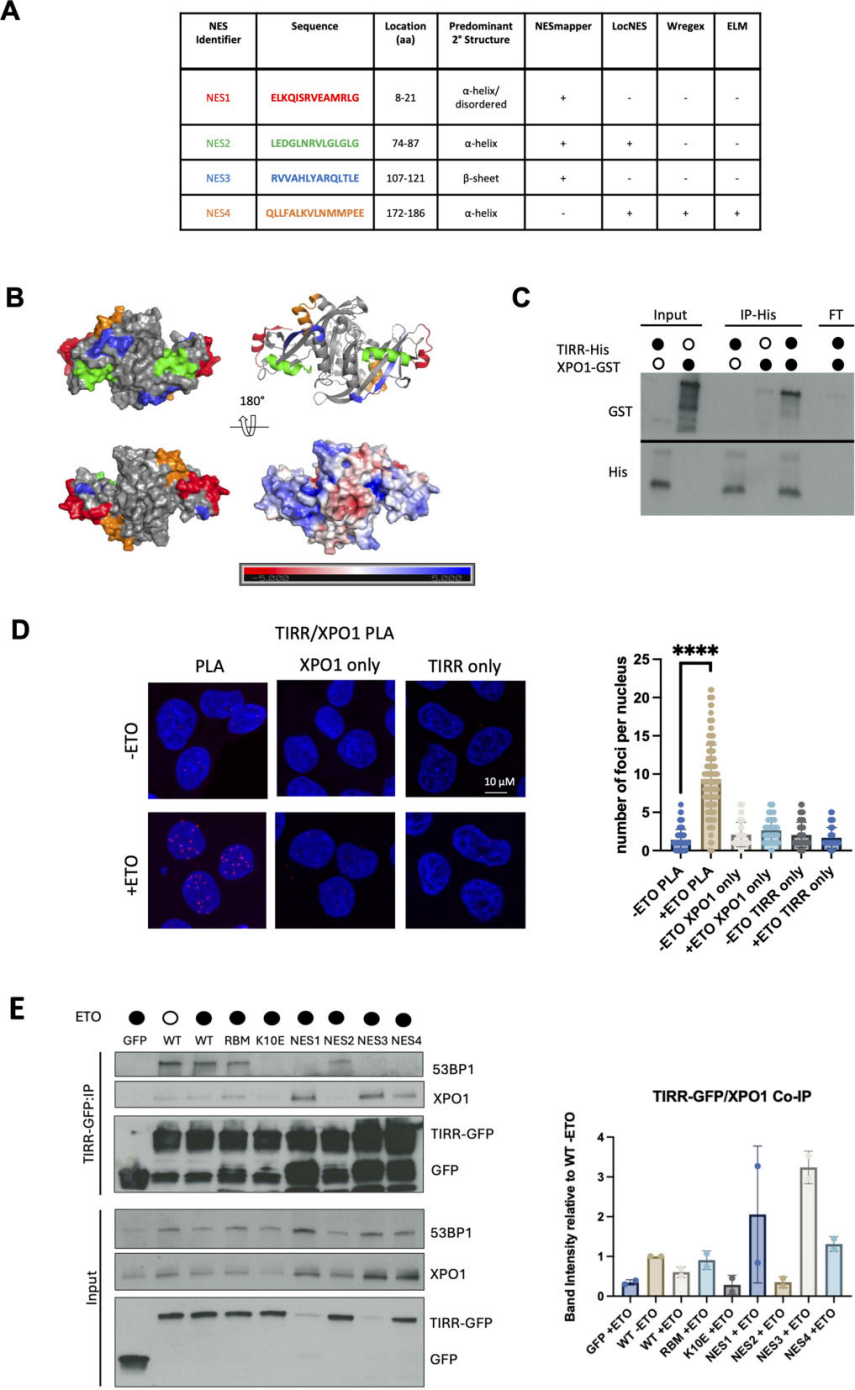
TIRR interacts with XPO1 upon DNA damage. (**A**) Table showing predicted NES on TIRR. (**B**) TIRR dimer (PDB: 6D0L) with the location of each predicted NES highlighted on both monomers in corresponding colour to (A). The lower right structure shows the electrostatic landscape of TIRR, with the region displaying increased positive charge (darker blue) in the clefts indicating the RNA binding regions of the dimer. (**C**) *In vitro* interaction assay with purified TIRR-His and recombinant XPO1-GST. Immunoblot showing GST and His signals. (**D**) Left: Confocal images showing PLA of TIRR and XPO1 in cells with or without ETO treatment. Single antibodies were used as a negative control. Right: Quantification of PLA signals. Significance was determined using a non-parametric Mann–Whitney test (****P ≤ 0.0001). (**E**) Western blot of co-IP of XPO1 and 53BP1 with TIRR-GFP WT in damage (+ETO) and non-damage conditions and TIRR-GFP mutants [RBM, 53BP1 binding mutant (K10E), NES mutants (NES1–NES4)] in damage conditions. A GFP-only control in damage was used to assess non-specific binding of XPO1 to the beads. Semi-quantification of XPO1 and 53BP1 band intensity on right. XPO1 pulldown band intensity was normalized first to GFP IP bands, then to -ETO WT.

Having established that XPO1 regulates export of TIRR to the cytoplasm upon DNA damage, we endeavoured to determine whether there were any NES in TIRR’s sequence that might facilitate the interaction between TIRR and XPO1. To address this question, we used several programmes designed to identify both general short amino acid motifs recognized by protein interactors [eukaryotic linear motif (ELM) and Wregex] and NES specifically (NESmapper and LocNES) (45–48). Combined, ELM, Wregex, NESmapper and LocNES identified four candidate NES motifs in TIRR (Figure 4A). The positions of these candidate NES in TIRR have been given an identifier (NES1, NES2, NES3 and NES4) (Figure 4B).

Before evaluating which of these NES may be responsible for XPO1 interaction, we first assessed whether we could detect a physical association of TIRR with XPO1. We incubated recombinant His-tagged TIRR (8) and GST-tagged XPO1, performed pulldown of TIRR and assessed for XPO1 binding. XPO1 was able to bind to TIRR in this assay, with minimal background binding of XPO1 detected on the beads in the absence of TIRR (Figure 4C). We then confirmed the interaction of TIRR and XPO1 through co-IP and western blot in both non-damage and damage conditions. Co-IP was performed in both ways, pulling down either TIRR-GFP (doxycycline-induced overexpression) or XPO1-FLAG (transient transfection). XPO1 co-IPed with TIRR-GFP in both damage and non-damage conditions, while no XPO1 was observed in a GFP-only control (Supplementary Figure S10B). TIRR also co-IPed with XPO1-FLAG in both non-damage and damage conditions, but not with mScarlet-FLAG (Supplementary Figure S10C and D). We next employed the quantifiable technique PLA, which is antibody based and allows for the detection of proximity between two proteins of interest in cells. PLA also allows the detection of weak and transient interactions. Using PLA with TIRR and XPO1 antibodies, we were also able to detect an interaction between TIRR and XPO1, which was significantly increased after DNA damage induced with either ETO (Figure 4D; single antibodies were used as a negative control) or IR (Supplementary Figure S10A). The discrepancy between co-IP and PLA may be explained by the tendency for PLA to provide a seemingly more exaggerated difference between treatment conditions (49). Additionally, the degree of interaction between TIRR and XPO1 increasing upon damage may not necessarily be the signifier of increased TIRR re-localization to the cytoplasm, as other contributions such as the binding of additional factors could play a role. Regardless, these data suggest that TIRR is an XPO1-interacting protein in the DDR.

We then generated GFP-tagged mutants of all the NES sites by alanine scanning of hydrophobic residues at each of the NES sites, a method employed in validating NES sites in previous studies (50,51). We also generated a GFP-tagged TIRR RNA-binding mutant (RBM) (27) and a GFP-tagged 53BP1 binding mutant (K10E) (4,5,52). Plasmids for transient transfection were initially generated (excluding K10E); however, expression across the various constructs was very uneven (Supplementary Figure S10E). Consequentially, stable U2OS cell lines were created with the full set of TIRR-GFP mutants, along with a GFP-only and a WT TIRR-GFP control. Expression was much more even across these cell lines, except for NES1 and NES3 that had significantly lower expression, perhaps due to toxic products (Supplementary Figure S10F). NES1 and NES3 are also located in the vicinity of the 53BP1 binding loop (4). PLA was used to validate the WT interaction of XPO1 with the stably expressed constructs, showing rescue of TIRR/XPO1 PLA foci in a KD background with WT TIRR-GFP, but not GFP alone (Supplementary Figure S10G). Co-IP was performed with the stable cell lines, adjusting the seeding density for NES1 and NES3 according to their reduced expression, and we found that mutation of the NES2 motif resulted in decreased TIRR binding to XPO1 (Figure 4E). Expression of GFP alone served as a negative control. This motif is located distant to the RNA binding groove. Mutations in NES1, NES3 and NES4 did not reduce TIRR binding to XPO1. Interestingly, XPO1 binding appeared to be reduced in the K10E 53BP1 binding mutant. A PLA between XPO1 and selected mutants (WT, NES2, NES4) was performed with transient transfection of the mutants and quantification of foci in cells with similar GFP expression, confirming reduced XPO1 binding in NES2 (Supplementary Figure 10H). Altogether, we conclude that TIRR binds the protein export factor XPO1 at least in part through direct interaction with its NES2 motif.

### TIRR associates with and regulates the formation of P-bodies in response to DNA damage

**Figure 5.**
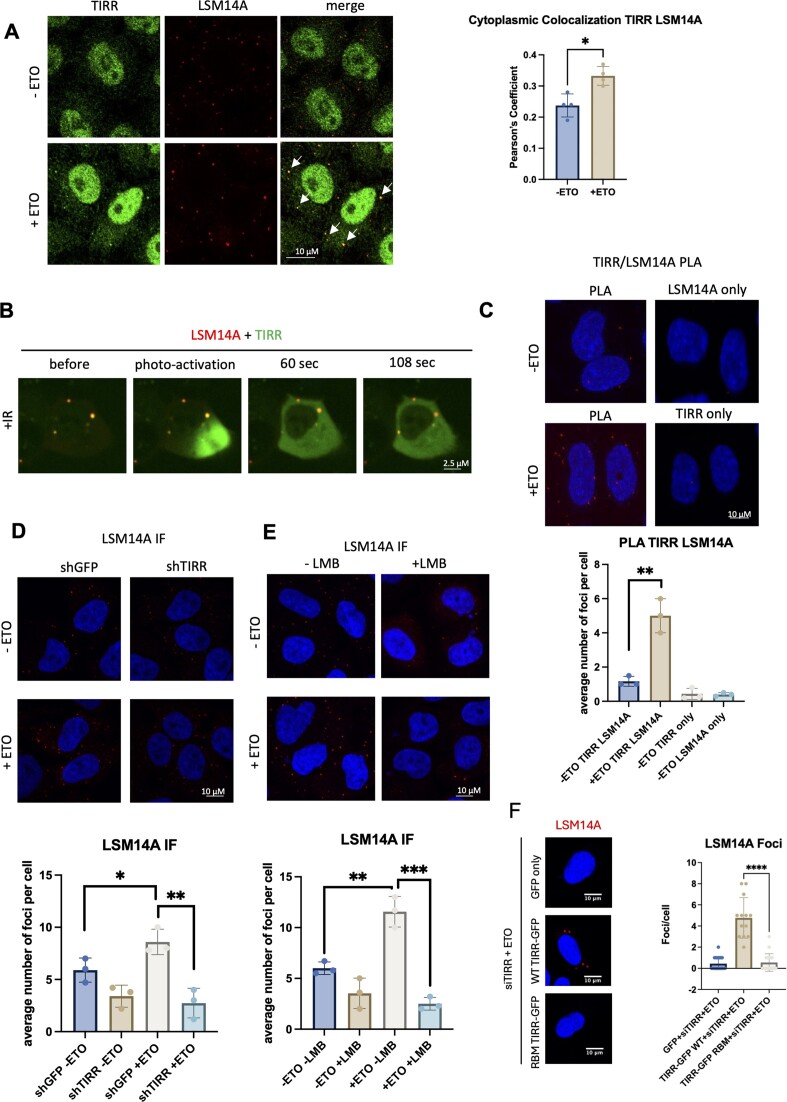
TIRR associates with and regulates P-body formation in response to DNA damage. (**A**) Left: Representative confocal images showing IF of TIRR in cells with or without ETO treatment. TIRR expression is visualized in the left panels, P-bodies are visualized with anti-LSM14A antibody in the middle panels, and a merge is provided in the right panels. White arrows point to P-bodies. Right: Quantification of TIRR and LSM14A co-localization signal in cytoplasm using Pearson’s coefficient (*n* > 50 cells); significance was determined using a *t*-test (*P ≤ 0.05). (**B**) Representative confocal images showing live cells expressing PA-TIRR-GFP (cytoplasmic and nuclear) and LSM14A-mScarlet (foci in cytoplasm) as a marker for P-bodies. Photoactivation was induced using a 413 nm laser, and the location of activation is indicated with a white arrow. Photoactivation was followed by time-lapse microscopy; time points at 60 and 108 s are shown. See also Supplementary Movie S4. (**C**) Top: PLA using anti-TIRR and anti-LSM14A antibodies in non-damage (−ETO) or damage (+ETO) conditions in HeLa cells. DAPI was used to label nuclei. PLA signals are visible as foci. Single antibodies were used as negative controls. Bottom: Quantification of top. Error bar = mean ± standard deviation; significance was determined using a non-parametric Mann–Whitney test (**P ≤ 0.01). (**D**) Top: IF analysis showing LSM14A staining in non-damage (−ETO) and damage (+ETO) conditions in cells expressing either shGFP (control) or shRNA targeting TIRR (shTIRR). DAPI was used to stain nuclei. Bottom: Quantification of TIRR signal in cytoplasm (*n* > 50 cells); significance was determined using a *t*-test (**P ≤ 0.01, *P ≤ 0.05). (**E**) Top: IF analysis showing LSM14A staining in non-damage (−ETO) and damage (+ETO) conditions and with or without LMB treatment. DAPI was used to stain nuclei. Bottom: Quantification of TIRR signal in cytoplasm (*n* > 50 cells); significance was determined using a *t*-test (***P ≤ 0.001, **P ≤ 0.01). (**F**) Left: IF of LSM14A in TIRR KD with siTIRR and rescue with GFP, TIRR-GFP WT or TIRR-GFP RBM. Right: Quantification of LSM14A foci (*n* > 10 cells); significance was determined using a *t*-test (***P ≤ 0.001, **P ≤ 0.01).

Analysis of RIP-seq data revealed that TIRR binds preferentially to RNAs with a lower GC content (Figure 1D), which are enriched in P-bodies (39). Interestingly, PBs are considered sites of both RNA decay and storage, influencing the stability of specific RNAs (17,39). RNAs targeted to PBs appear to be related to regulatory processes such as RNAPII transcription, allowing PBs to dynamically influence the cellular response to specific stimuli (24). Therefore, we wondered whether TIRR and its bound RNA could be associated with PBs. To test this hypothesis, we performed co-IF of TIRR and LSM14A [LSM14A is an established marker of P-bodies and is essential for their formation (24,53,54)] using antibodies against endogenous proteins and observed distinct TIRR foci co-localizing with LSM14A foci. This co-localization was significantly increased upon DNA damage, as assessed by Pearson’s co-localization coefficient (Figure 5A). We also overexpressed TIRR in HeLa cells and performed co-IF using antibodies against GFP and LSM14A in cells subjected to IR. Distinct foci containing overlapping green (TIRR-GFP) and red (LSM14A) signals were detected in damaged cells (Supplementary Figure S11A). To test the interaction between TIRR and LSM14A in live cells, we transfected U2OS cells with PAGFP-TIRR and LSM14A-mScarlet and observed interaction between these two proteins in the form of overlapping foci that co-localized continuously within the tested time frame (Figure 5B and Supplementary Movie S4).

Finally, we employed PLA using antibodies against TIRR and LSM14A in HeLa and U2OS cells to assess whether their visual proximity was reflected in molecular proximity. Indeed, upon ETO-induced DNA damage in both cell lines, PLA foci number increased significantly, suggesting proximity (<∼40 nm) of TIRR and LSM14A after DNA damage (Figure 5C and Supplementary Figure S11B). TIRR and LSM14A antibody only in non-damage conditions were used as negative controls. Similarly, we observed damage-dependent PLA foci formation between TIRR and LSM14A in HeLa cells subjected to IR (Supplementary Figure S11C). Collectively, we have shown that TIRR associates with P-bodies, and this interaction is enhanced by DNA damage.

To further understand the significance of this interaction, we performed IF of LSM14A foci in shTIRR and shGFP HeLa cells with induction of DNA damage by ETO or IR. Upon DNA damage in control conditions, an increase in P-body number was observed when compared to non-damage. This agrees with previous studies of P-body formation, where it was found that various stresses and DNA damage conditions resulted in increased P-body formation (55–57). Interestingly, TIRR depletion resulted in decreased P-body formation (Figure 5D and Supplementary Figure S11D). LSM14A protein levels are not affected in TIRR KD cells (Supplementary Figure S4A), suggesting that TIRR is modulating P-body formation via a different mechanism. We also show that the overexpression of TIRR-GFP could elicit an increase in P-body numbers (Supplementary Figure S11E and F). These data suggest that TIRR can co-localize with P-bodies, particularly after damage, and can modulate P-body formation.

We showed that TIRR can interact with XPO1. Therefore, we assessed whether TIRR modulation of P-body numbers is dependent on XPO1 activity. We performed IF of LSM14A upon damage and inhibition of XPO1 with LMB. Indeed, P-body number was significantly reduced in DNA damage induced by ETO upon LMB treatment in HeLa or U2OS cells (Figure 5E and Supplementary Figure S12A). Similarly, this was also observed in HeLa cells subjected to IR (Supplementary Figure S12B). We hypothesized that the regulation of PB formation by TIRR may be through TIRR providing RNA as a scaffold for PB formation in the cytoplasm, and therefore we assessed whether PB formation was dependent on the RNA binding function of TIRR. We performed KD of endogenous TIRR and transient overexpression of either GFP alone, TIRR-GFP WT or TIRR-GFP RBM. While TIRR-GFP WT rescued PB formation, cells with similar GFP expression levels expressing TIRR-GFP RBM had dramatically fewer PBs (Figure 5F). Therefore, we conclude that TIRR can regulate the formation of P-bodies, and this function is dependent on its RNA binding function, as well as XPO1 export.

### TIRR-bound mRNAs are associated with P-bodies

**Figure 6.**
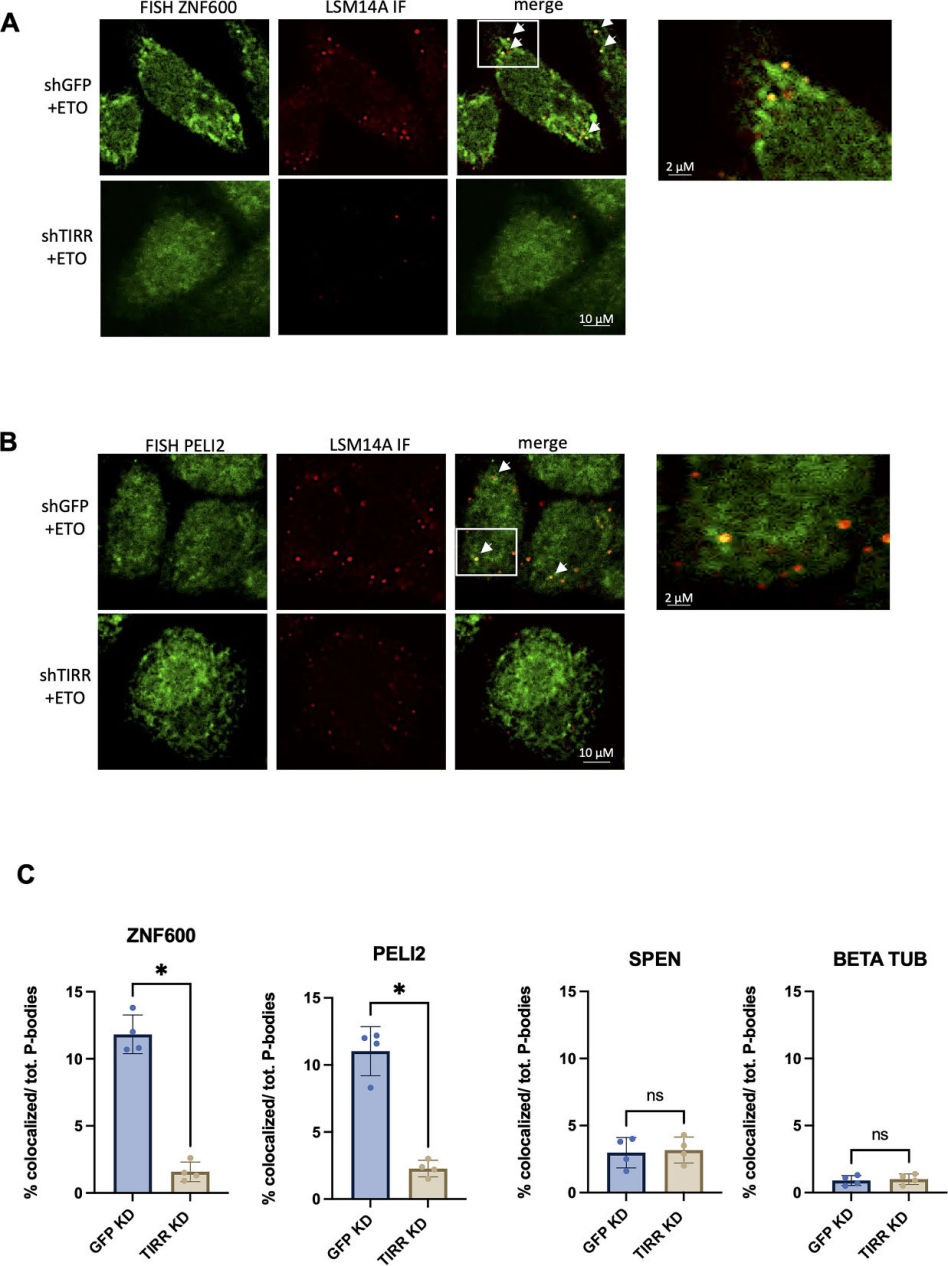
TIRR depletion leads to accumulation of mRNA in nucleus. (**A**) Representative confocal images showing co-localization of RNA FISH signals (left panel, merged with PB foci on right) for *ZNF600* with LSM14A (PB foci, middle panel) in shGFP or shTIRR cells after ETO treatment (*n* > 50 cells). White rectangles mark zoomed-in images on the right. White arrows point to P-bodies with FISH signal. (**B**) As in panel (A), for *PELI2* mRNA. (**C**) Quantification of FISH and LSM14A co-localization signal in *ZNF600*, *PELI2*, *SPEN* and *Tubulin* by calling of PB foci with FISH signal overlap (*n* > 50 cells); significance was determined using a *t*-test (*P ≤ 0.05, **P ≤ 0.01).

We detected TIRR being associated with P-bodies (Figure 5) and therefore we wished to investigate whether TIRR-bound mRNA is also localized to PBs. First, we performed RNA FISH in the presence or absence of ETO with probes against *ZNF600* and *PELI2*, two mRNAs found to bind to TIRR specifically upon damage in our RIP-seq, as well as probes against *SPEN*, an mRNA known to localize to P-bodies but not found in our RIP-seq, and *Tubulin*, an mRNA also not found in our RIP-seq (24). This was followed by IF co-staining with antibody for LSM14A in HeLa cells expressing either shTIRR or shGFP. Close analysis of confocal images revealed assembly of *ZNF600* or *PELI2* FISH probes in cytoplasmic foci (green) that overlapped with LSM14A (red) IF signals (Figure 6A–C). Overlapping foci were called by identifying yellow foci in the merged images. This co-localization was abolished in TIRR KD cells, most likely due to impaired P-body formation and mRNA nuclear retention (Figure 6A–C). Additionally, *SPEN* co-localization with P-bodies in damage conditions remained apparent and was not affected by depletion of TIRR, while *Tubulin* had no apparent co-localization with TIRR in any condition (Supplementary Figure S13A and B). FISH–LSM14A foci overlap with *ZNF600* or *PELI2* were also observed in cells subjected to IR (Supplementary Figure S13C and D).

These data show that specific TIRR-bound mRNAs are localized in PBs upon DNA damage in a TIRR-dependent manner.

## Discussion

The RNA binding function of TIRR, particularly in the DDR, remains largely unexplored. In this study, we demonstrate that TIRR exhibits binding affinity towards a specific subset of mRNAs in response to DNA damage and non-damage (Figure 1A–C). Notably, these mRNAs exhibit a significantly lower GC content compared to the rest of the genome (Figure 1D). We identify that selected damage-specific TIRR-bound RNA is dependent on TIRR for localization to the cytoplasm, especially upon DNA damage (Figure 2). TIRR localizes to the cytoplasm upon DNA damage, and its cytoplasmic localization is regulated by LMB, an XPO1 inhibitor (Figure 3). We show that TIRR interacts with XPO1 after DNA damage (Figure 4) and identify a specific binding motif, NES2, required for direct binding to XPO1. Interestingly, an established 53BP1 binding mutant (K10E) also shows reduced XPO1 binding (Figure 4E and Supplementary Figure S14A). While not further explored, this poses the question of a potential relationship between XPO1 and 53BP1 in TIRR binding.

Much like other NUDIX-related proteins (56), TIRR can localize to P-bodies, particularly following DNA damage, and is capable of regulating PB formation (Figure 5). Considering the observed impact of TIRR on PB formation, we propose that TIRR selectively binds to a subset of mRNAs to facilitate their sequestration into PBs. Consistent with this hypothesis, an analysis of RNAs enriched in PBs revealed their involvement in regulatory pathways that necessitate dynamic regulation, such as RNA processing, cell division and RNAPII transcription, similar to those found to be bound to TIRR upon DNA damage (Supplementary Figure S2A) (24,57). Our data demonstrate that TIRR localizes a subset of mRNAs into PBs, particularly after DNA damage (Figure 6).

Analysis of RNA-seq did not reveal large changes in total RNA levels in response to TIRR KD and ETO at the steady-state level, nor did we detect significant changes in protein product levels of several transcripts bound to TIRR upon damage. This is not necessarily unexpected, given that TIRR appears to sequester this RNA to P-bodies. Microscopically visible P-bodies have not conclusively been demonstrated to be required for activities such as mRNA decay and miRNA-mediated translational silencing, nor does targeting of an mRNA to P-bodies necessitate degradation (20,53,58). Yeast cells deficient in *lsm4* and *edc3* genes do not form P-bodies, and yet still show mRNA decay activity (59). It has been posited that P-bodies may only form due to the accumulation of translationally repressed mRNA complexes, but do not themselves instigate decay or silencing (53).

We show that TIRR binds to mRNAs involved in dynamic regulatory processes, such as transcription, particularly after DNA damage (Supplementary Figure S2A). Part of the cellular response to DNA damage is decreased transcription (60). Therefore, it is possible that TIRR selectively sequesters mRNA transcription factor transcripts to RNA granules for their storage, indirectly contributing to DSB-induced transcriptional repression. Furthermore, other roles of P-bodies have been suggested, including a storage function for inactive mRNA decay factors and translationally repressed transcripts and/or maintenance of the stability of localized mRNA through mechanisms such as polyA protection (59,61). As we have not directly assessed decay intermediates of TIRR-bound mRNA in DNA damage, we cannot exclude that some TIRR-mediated modulation of mRNA metabolism is occurring. Beyond P-body-mediated processes, in our model, these mRNAs originate in the nucleus, where decay of mature mRNA transcripts and translation largely do not occur (62,63). It is therefore within expectations that RNA and protein levels would remain unchanged during nuclear retention after TIRR KD.

From an alternative perspective, export of TIRR and its bound RNA from the nucleus may be the critical step of the pathway, rather than sequestration of RNA to P-bodies. Studies in fertilized *Xenopus laevis* eggs showed that depletion of Gle1, a cofactor required for efficient Ddx19-mediated mRNA export, or Nxf1, a factor associated with the export of mRNA, induced DNA damage as detected by γ-H2AX foci (64,65). DNA damage induced by a DNA topoisomerase I inhibitor (camptothecin) was at least partially dependent on mRNA export for resolution. Initial experiments suggest that XPO1 inhibition in non-damage conditions (Supplementary Figure S14C) results in a significant increase in γ-H2AX intensity in the nucleus, a marker of unresolved DSBs, suggesting that nuclear export by XPO1 may be important for resolving basal levels of DSBs. We also find a significant, however subtle, reduction in DNA damage clearance efficiency in endogenous TIRR KD, rescued with NES2 mutant, as detected by nuclear γ-H2AX intensity (Supplementary Figure S14B, D and E). This reduction is comparable to the reduction of clearance efficiency found in rescue with the 53BP1 binding mutant K10E, which is established as having reduced clearance efficiency (5). It is difficult at this stage to separate how much loss of XPO1 binding or 53BP1 binding contributes to this phenotype. Additionally, the degree of overexpression of the WT and mutant constructs may reduce the strength of the phenotypes. These findings introduce an intriguing novel method by which TIRR can influence DNA damage repair through export by XPO1.

Overall, here we identified TIRR as a regulator of mRNA localization in response to DNA damage. TIRR selectively binds to a subset of mRNAs encoding for proteins involved in transcription regulation, relocates to the cytoplasm through XPO1 and sequesters RNA into P-bodies. These findings extend our understanding of RNA regulation upon DNA damage.

## Supplementary Material

gkae688_Supplemental_Files

## Data Availability

All data are available in the paper and the supplementary data. The raw and processed RIP-seq and RNA-seq datasets described in this manuscript are deposited under Gene Expression Omnibus accession number GSE227686 (https://www.ncbi.nlm.nih.gov/geo/query/acc.cgi?acc=GSE227686).
