# Supplementary material for: TIRR regulates mRNA export and association with P-bodies in response to DNA damage
Source: Nucleic Acids Res. 2024 Aug 9;52(20):12633–49. doi: 10.1093/nar/gkae688 (PMC11551748; doi:10.1093/nar/gkae688)
Supplement: gkae688_Supplemental_Files [file gkae688_supplemental_files.zip › Supplementary Files TIRR mRNA Export.pdf]

Supplementary Figure 1

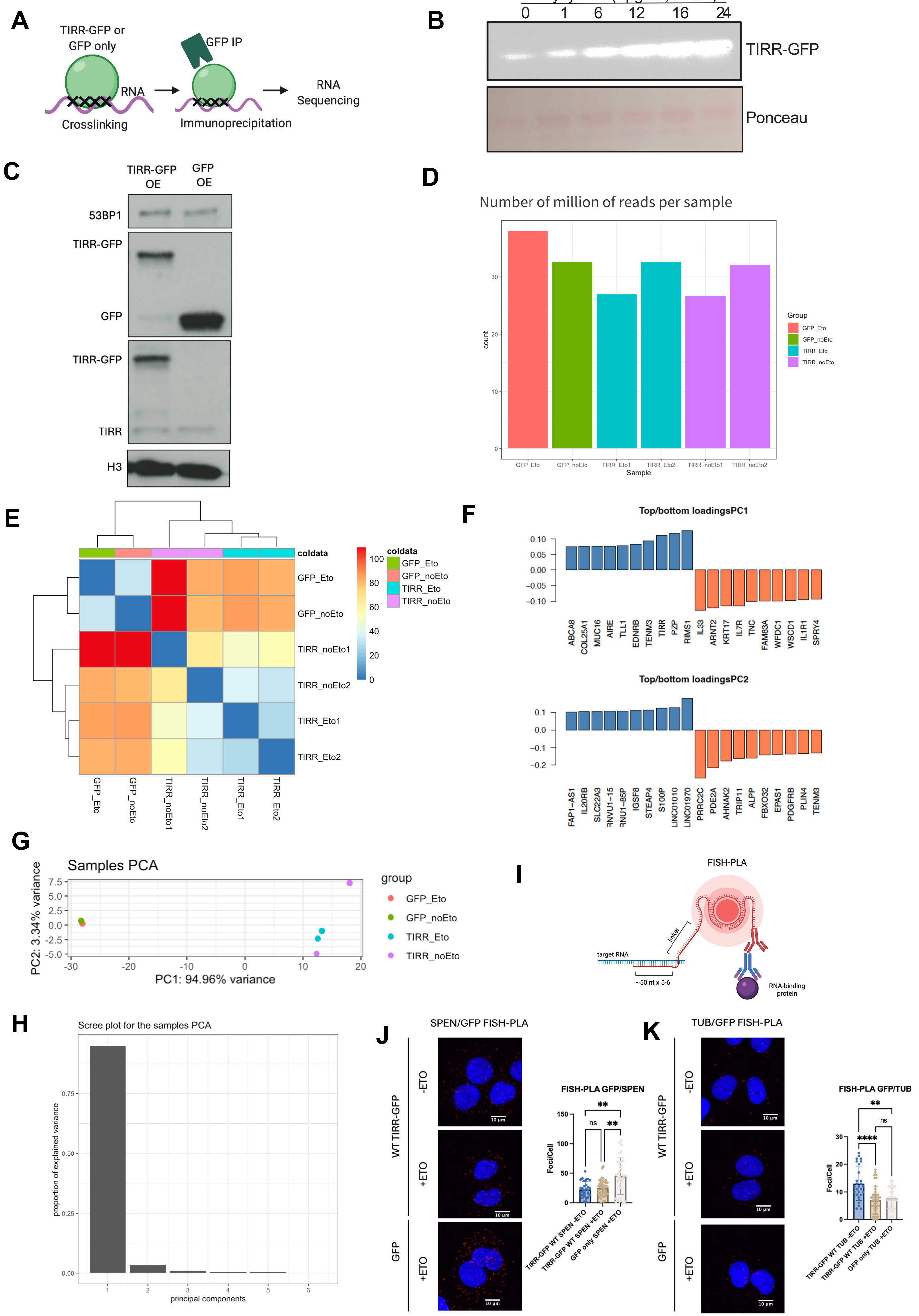

Supplementary Figure 2

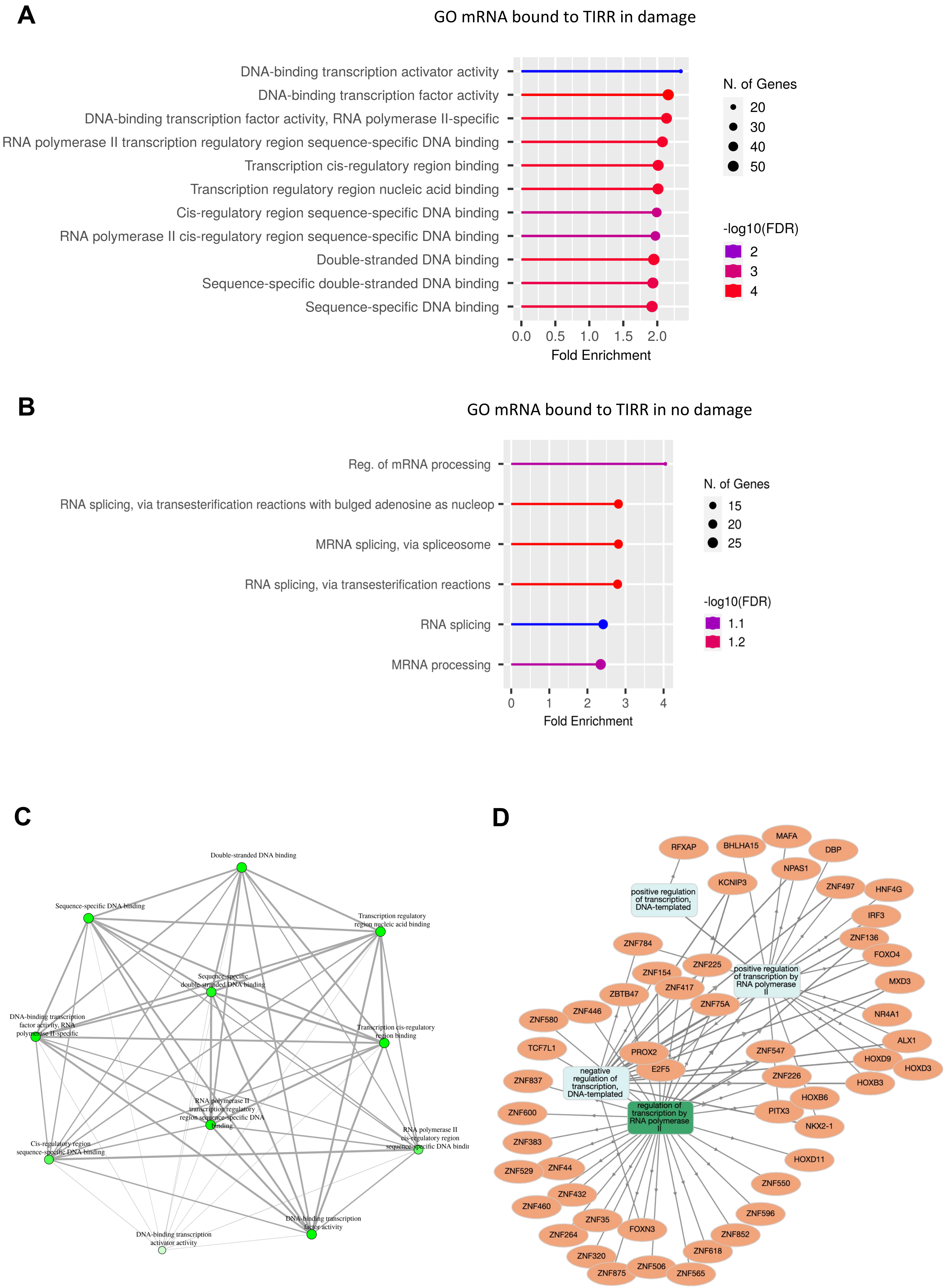

Supplementary Figure 3

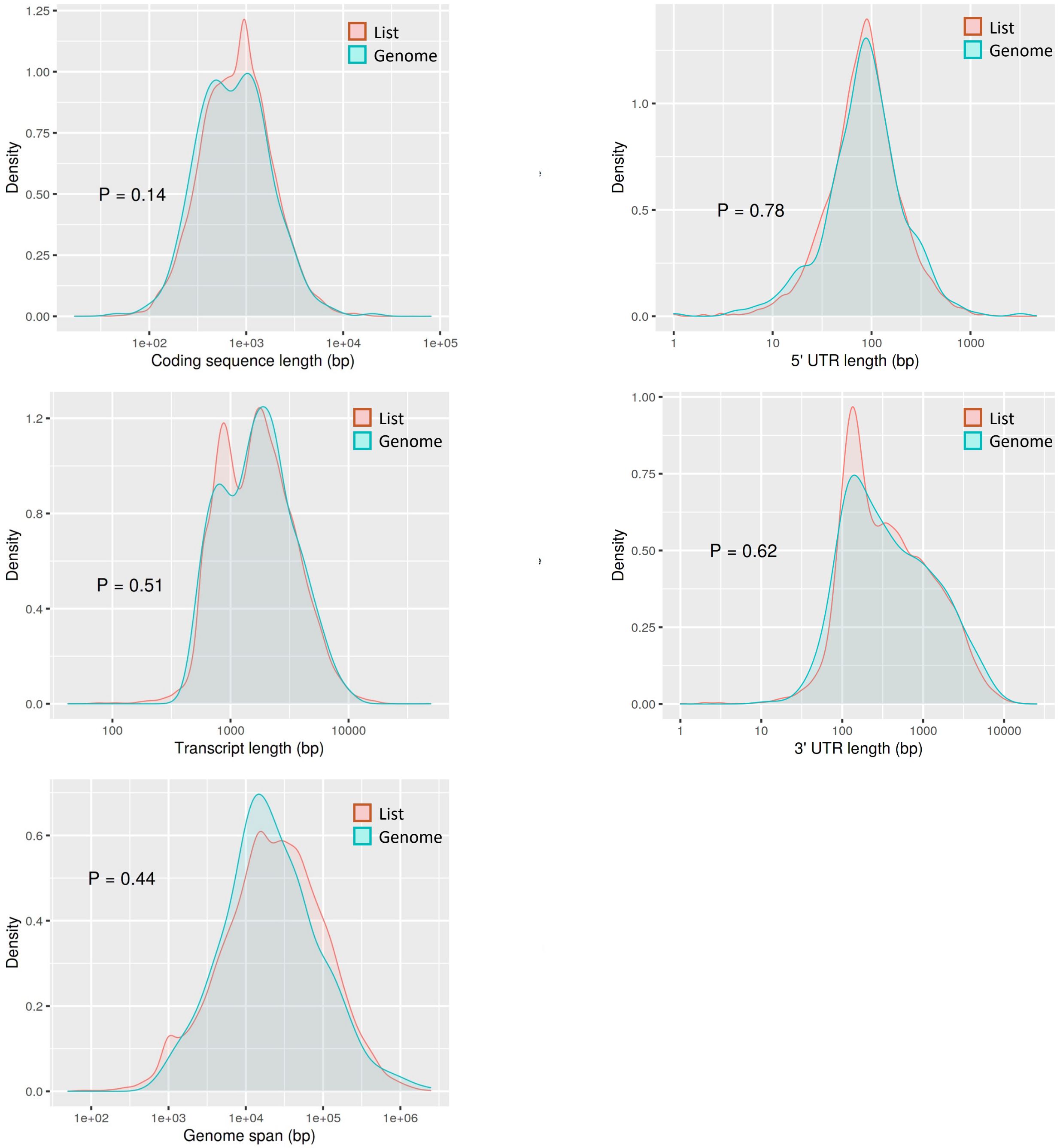

Supplementary Figure 4

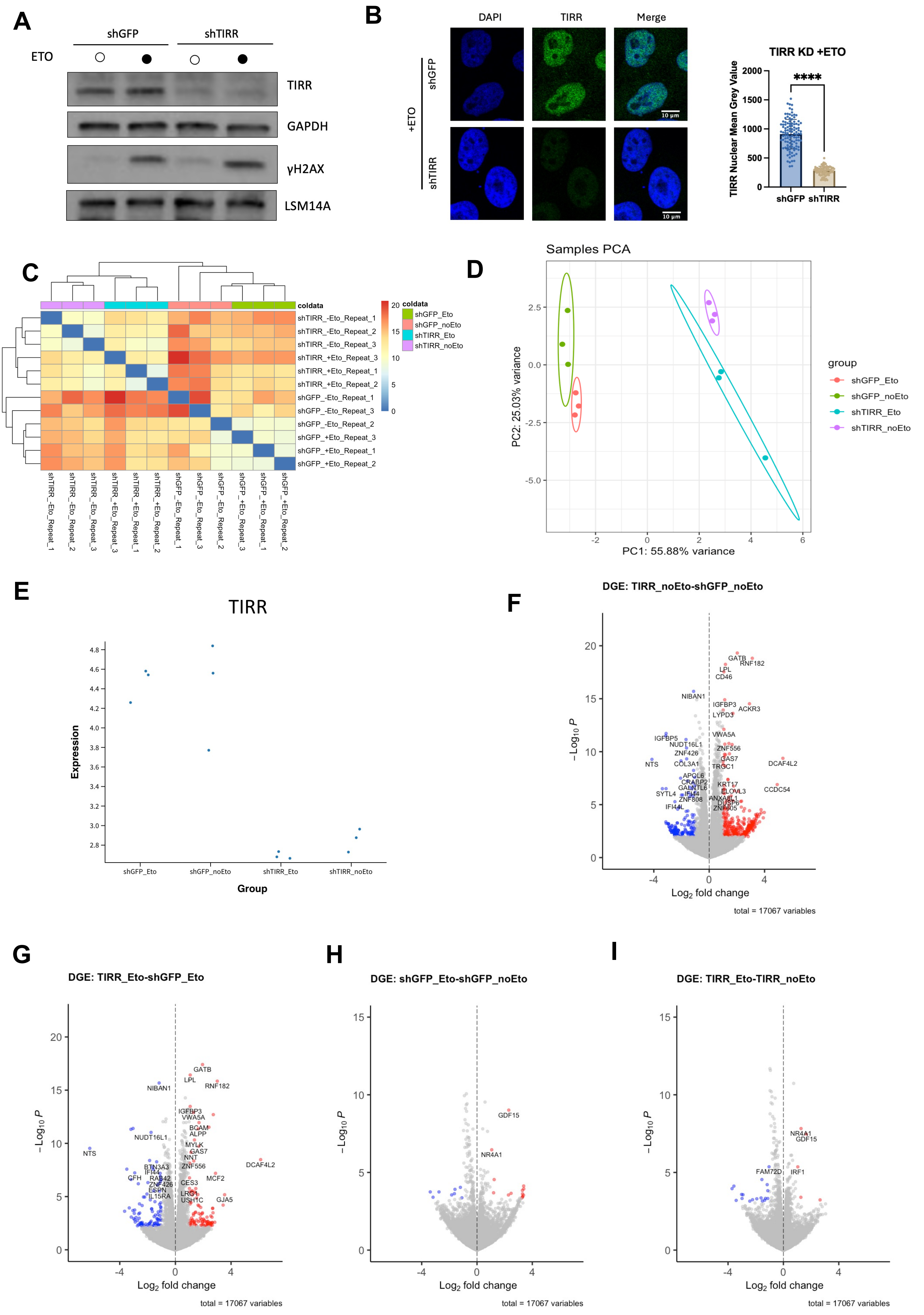

Supplementary Figure 5

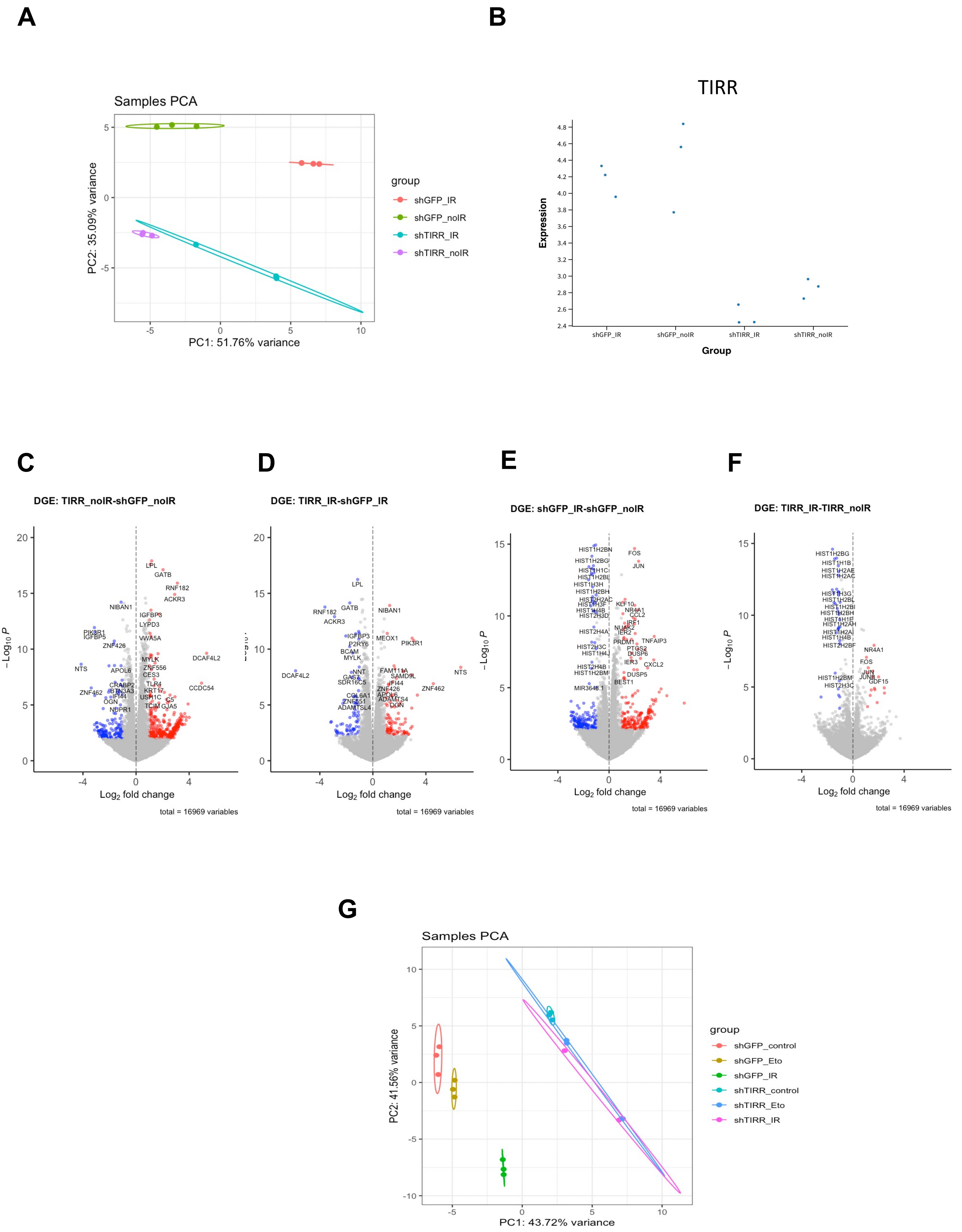

Supplementary Figure 6

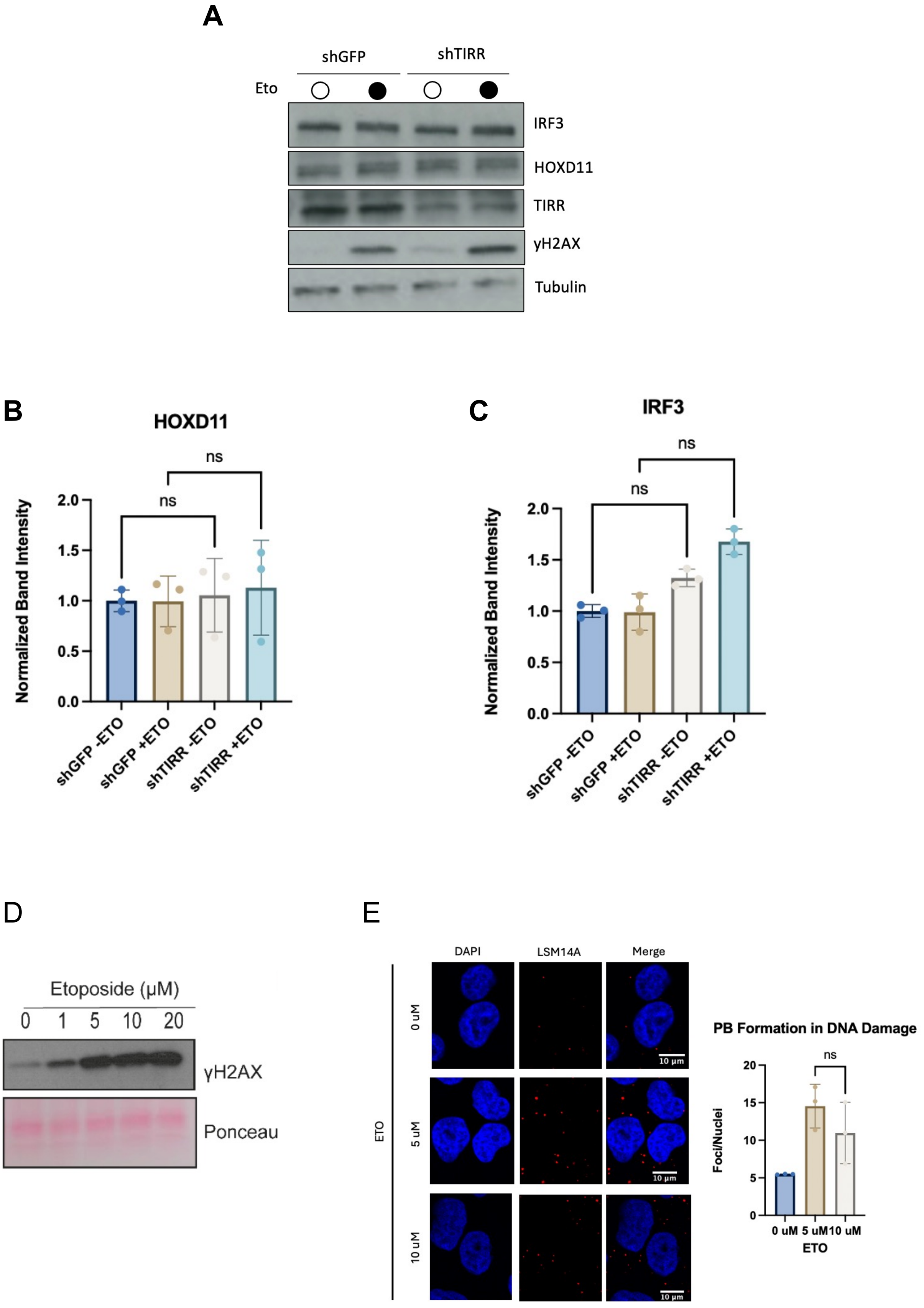

Supplementary Figure 7

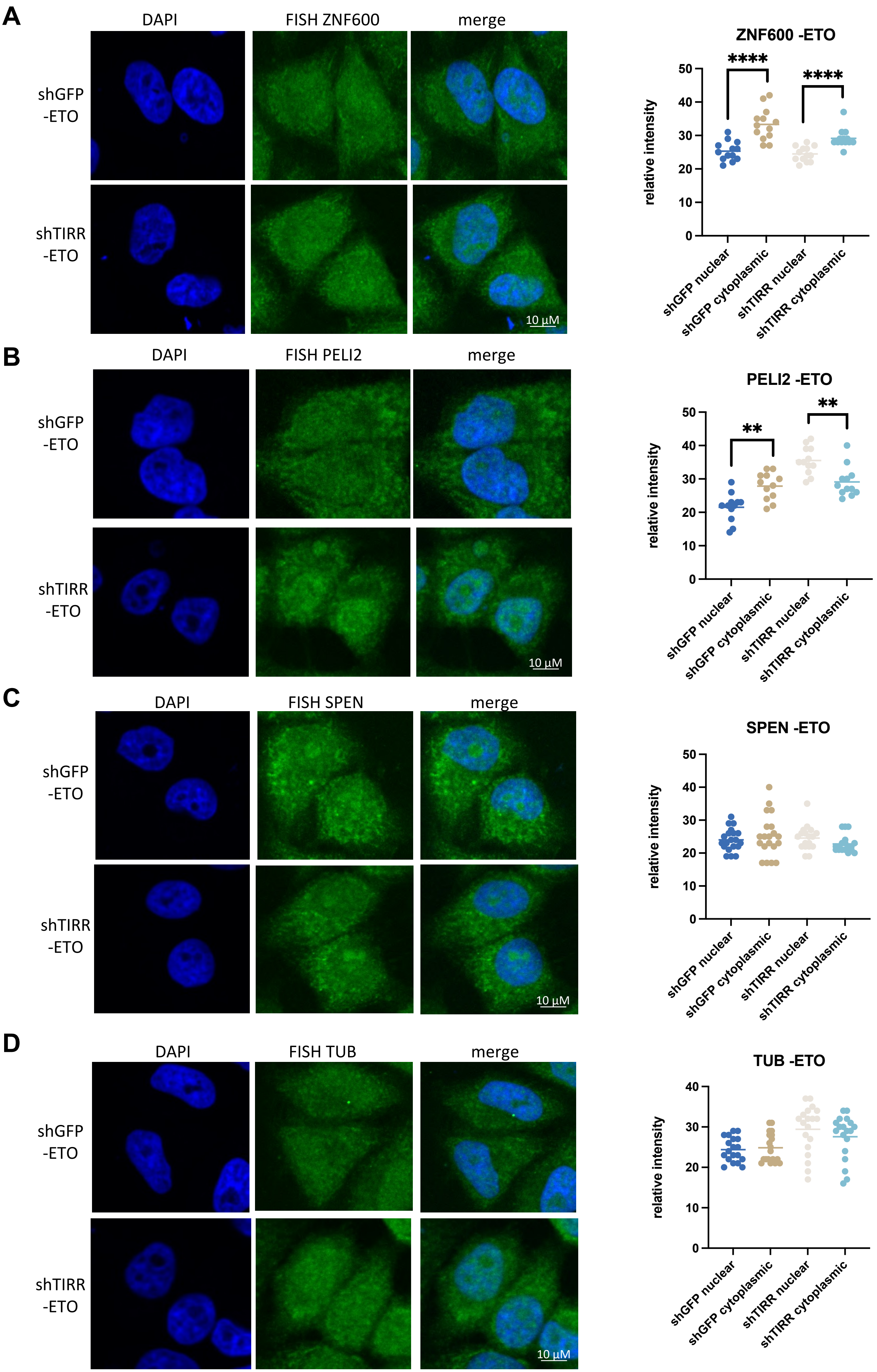

Supplementary Figure 8

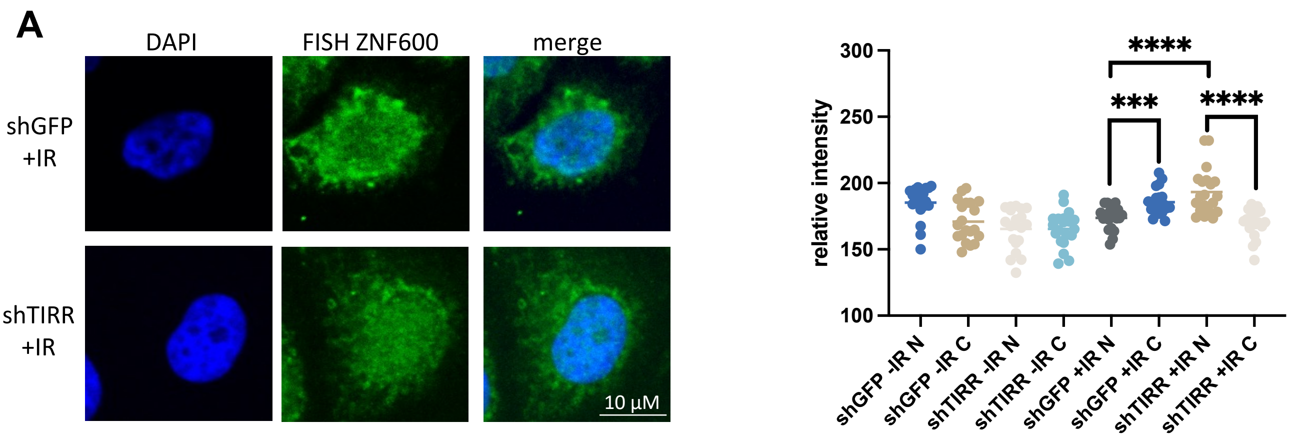

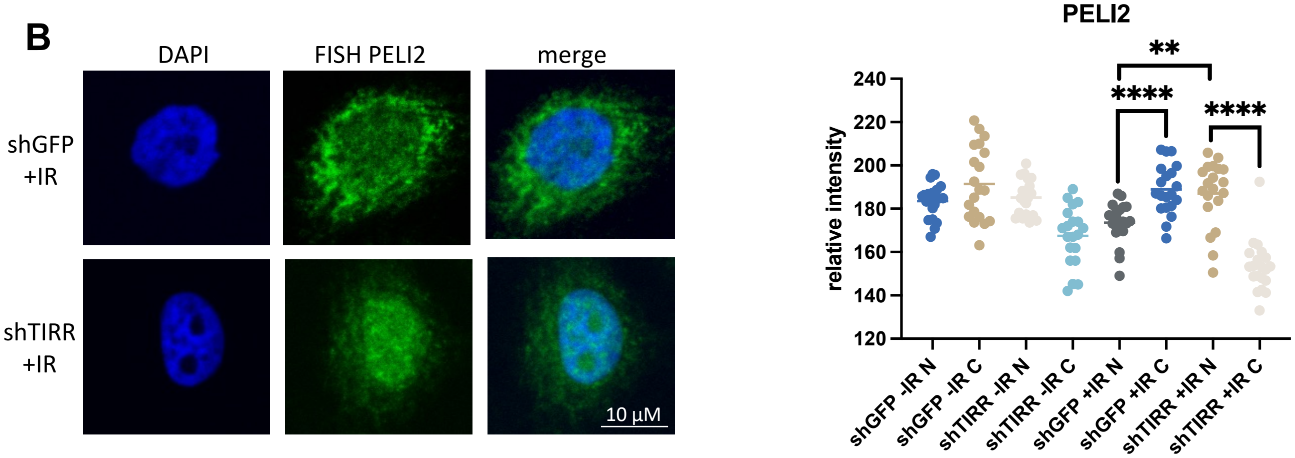

Supplementary Figure 9

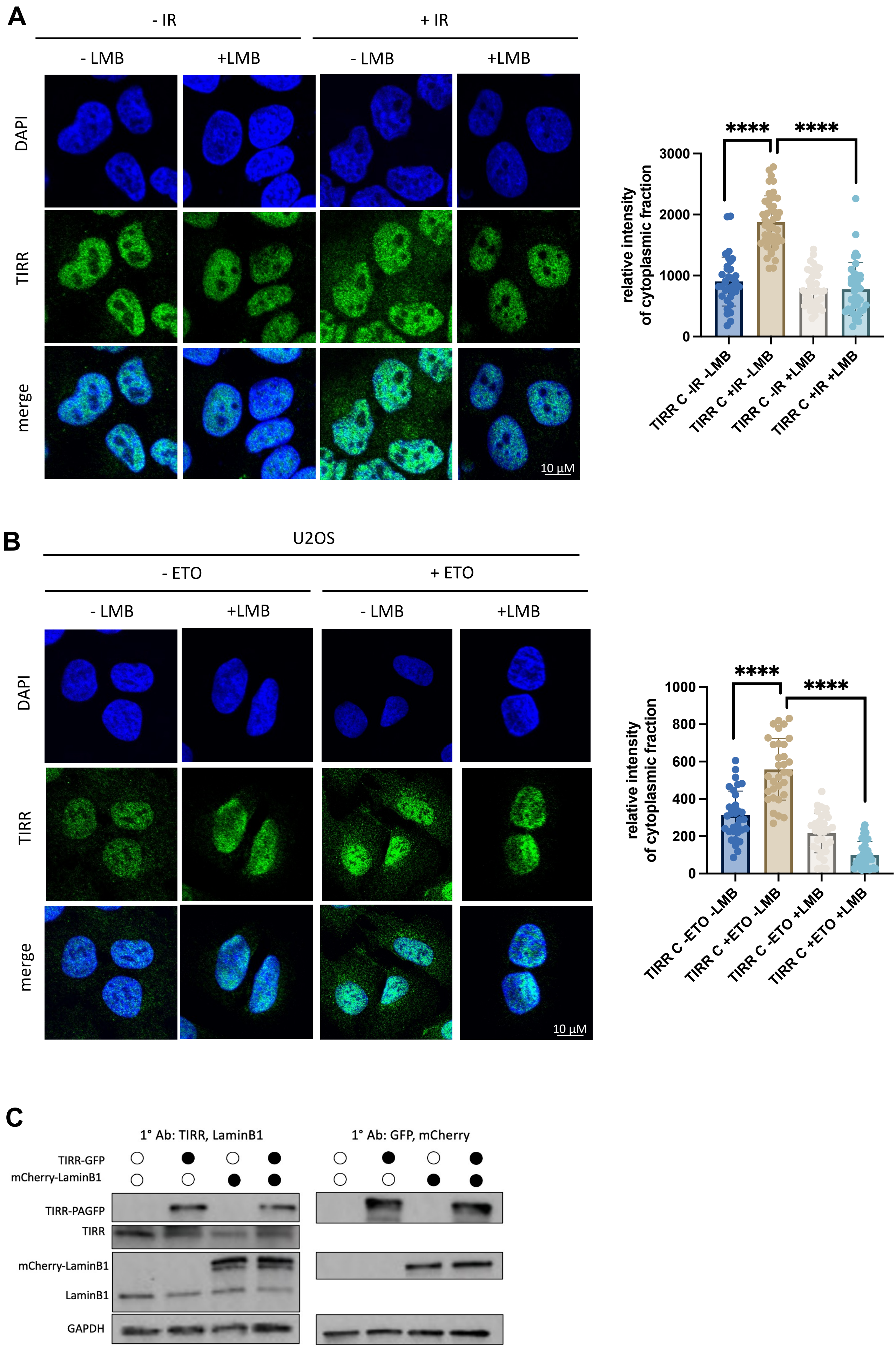

Supplementary Figure 10

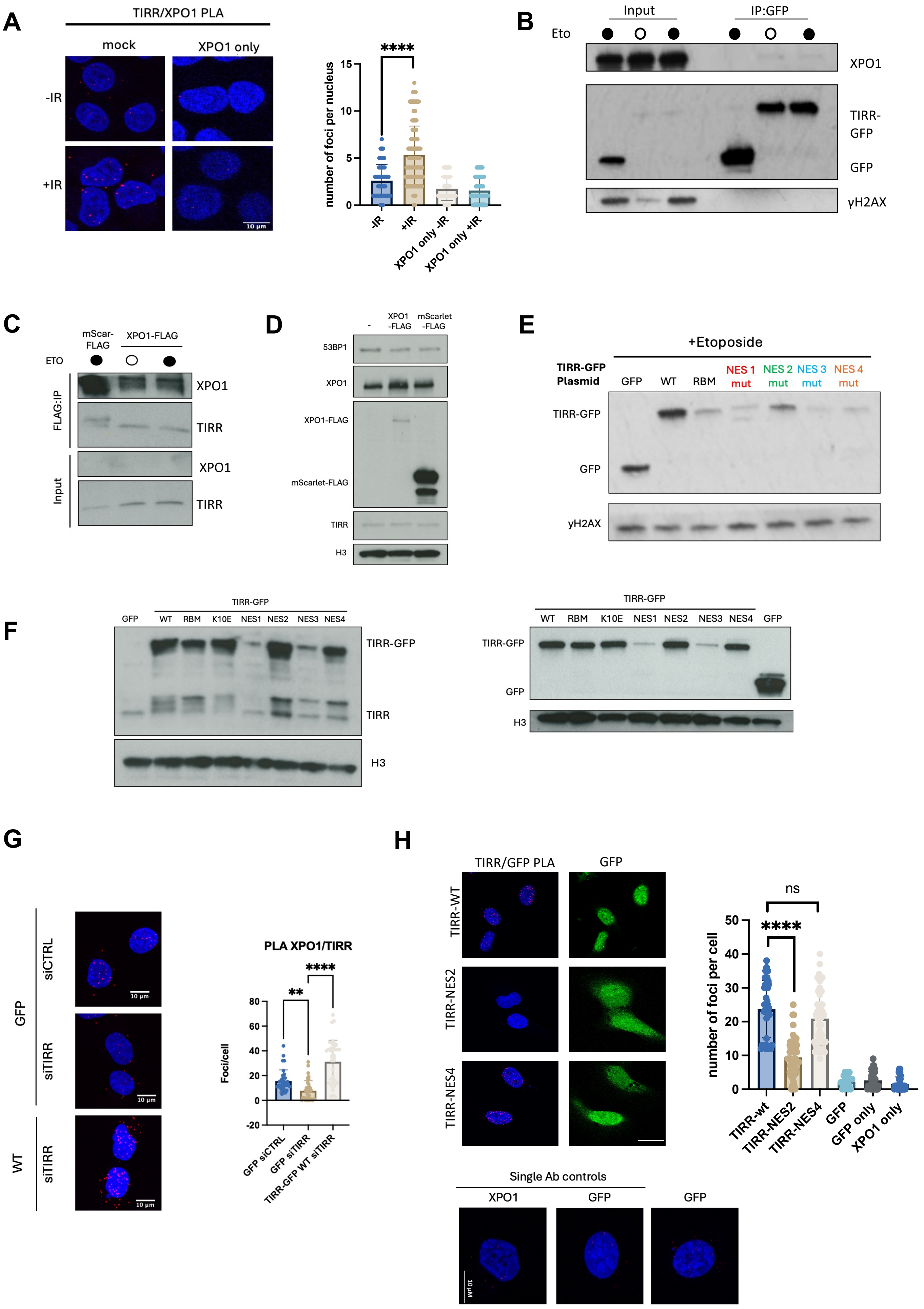

Supplementary Figure 11

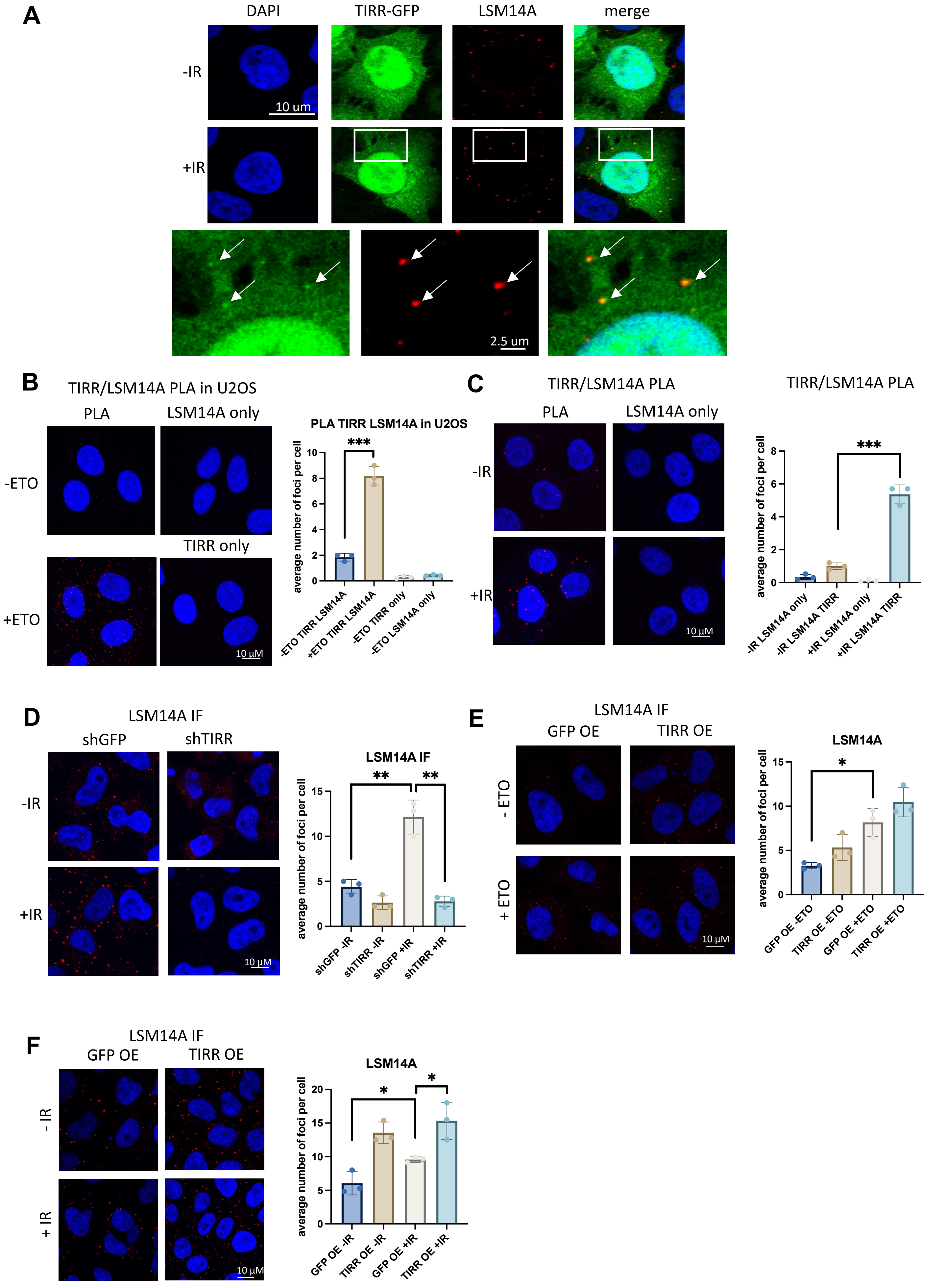

Supplementary Figure 12

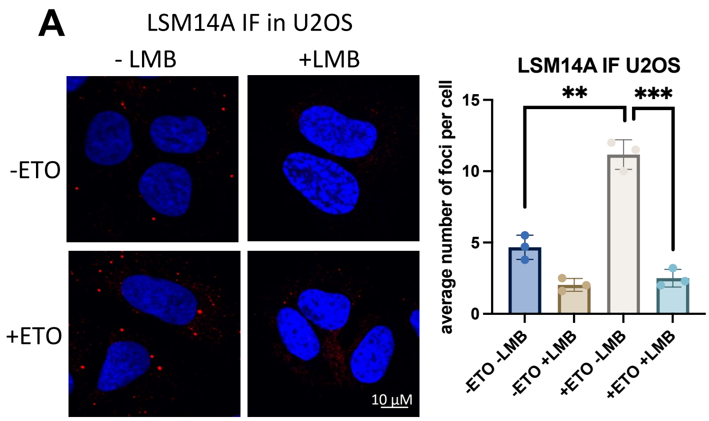

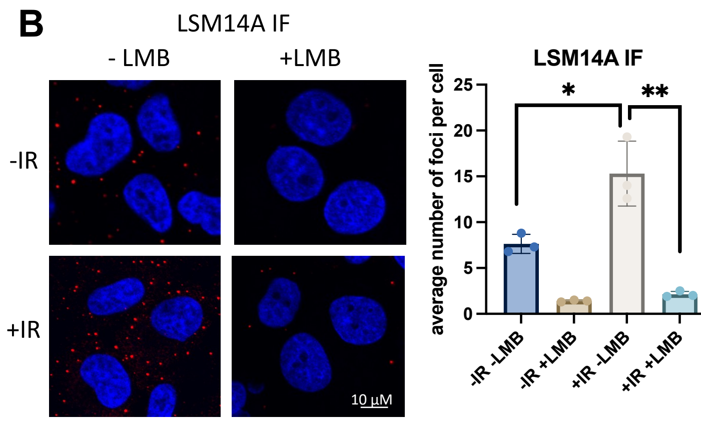

Supplementary Figure 13

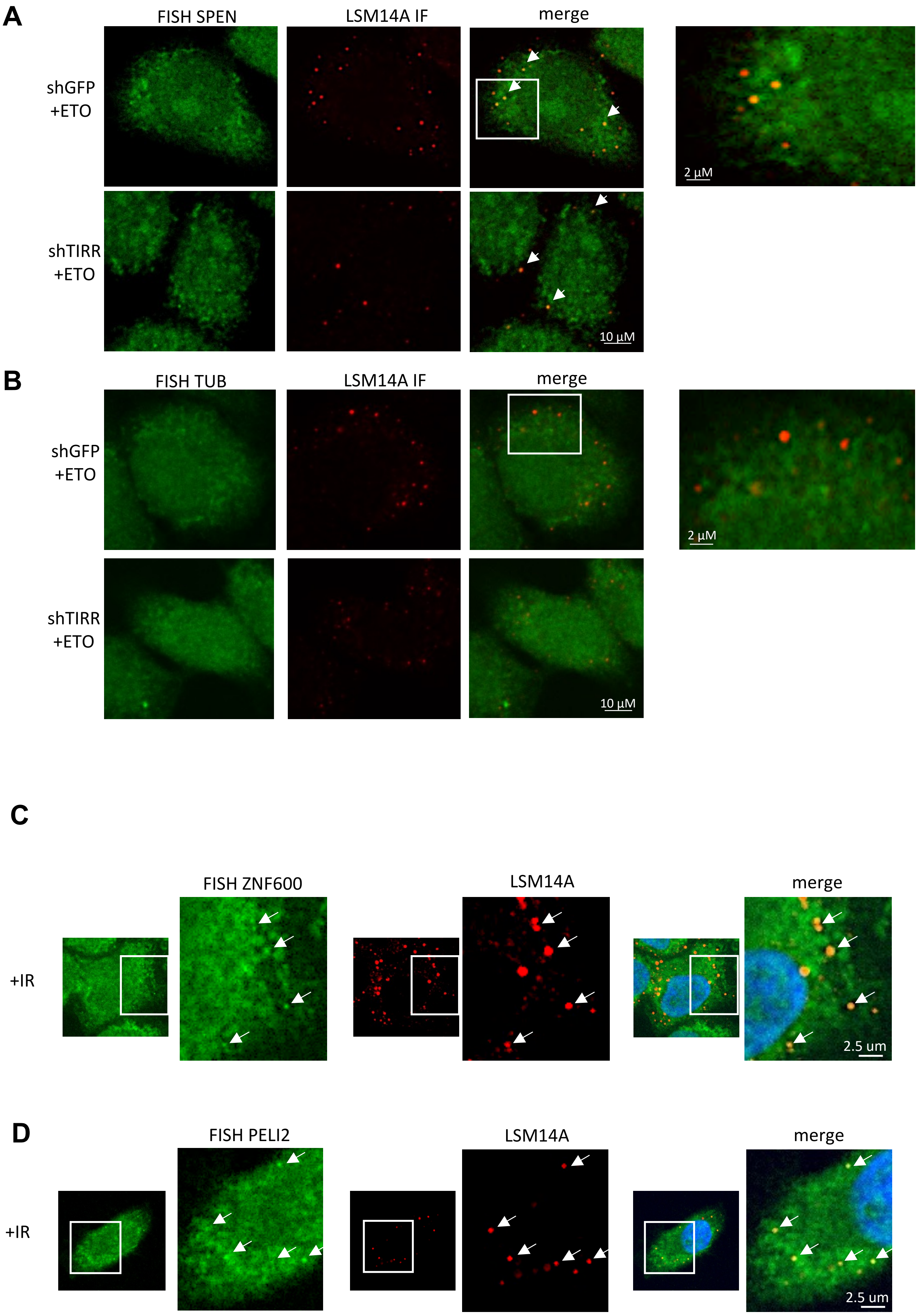

Supplementary Figure 14

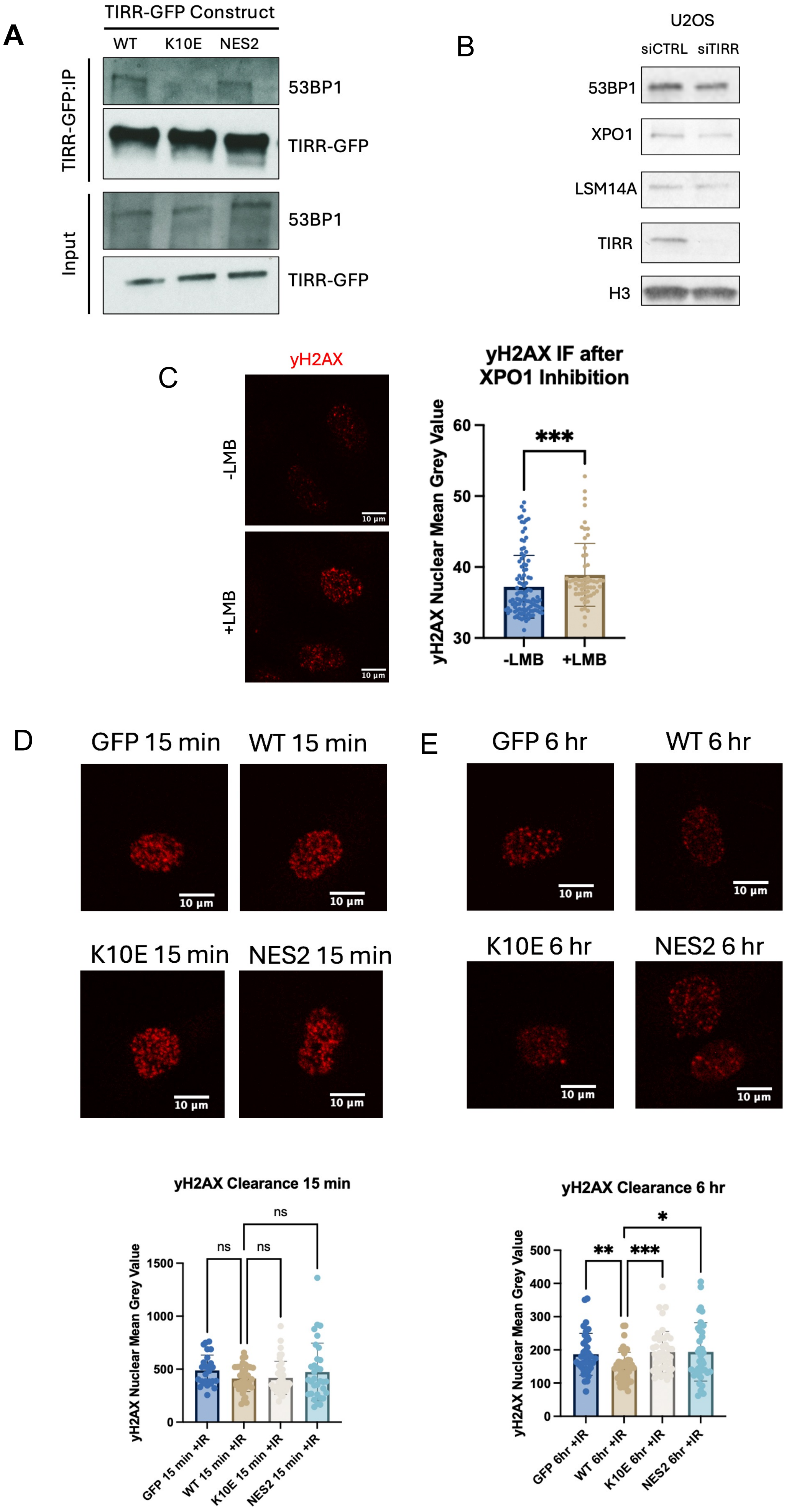

Supplementary Table 3

Oligonucleotide Sequences

Oligo	Sequence	Use	Modification
PELI2 FISH PROBE	AAGCACCATTGTACCCGAGC	FISH	5' Alexa Fluor NHS ester
ZNF600 FISH PROBE	TGCGTTTGGAAGAGATATCCAC	FISH	5' Alexa Fluor NHS ester
SPEN FISH PROBE	AGCGCTCCAAACGAGACTTG	FISH	5' Alexa Fluor NHS ester
BETA-TUBULIN FISH PROBE	CAGAGTCCATGGTCCCAGGT	FISH	5' Alexa Fluor NHS ester
TIRR_BACK_FWD	TGGACGAGCTGTACAAGTAACAATTCACTCGATCGGCTCGCTGATC	Gibson Cloning PAGFP-TIRR (TIRR backbone)	None
TIRR_BACK_REV	AGCTCCTCGCCCTTGCTCACCTCGACATCGATTGCGGCCGCAGAG	Gibson Cloning PAGFP-TIRR (TIRR backbone)	None
PA-GFP_FWD	CGGCCGCAATCGATGTCGAGGTGAGCAAGGGCGAGGAGCTGTTCA	Gibson Cloning PAGFP-TIRR (PA-GFP fragment)	None
PA-GFP_REV	CGAGCCGATCGAGTGAATTGTTACTTGTACAGCTCGTCCATGCCG	Gibson Cloning PAGFP-TIRR (PA-GFP fragment)	None
MSCARLET_FWD	CGCTACCGGTCGCCACCATGGTGAGCAAGGGCGAGGCAGTGATCAAG	Gibson Cloning LSM14A-mScarlet (mScarlet fragment)	None
MSCARLET_REV	TTGAGCTCGAGATCTGAGTACTTGTACAGCTCGTCCATGCCGCCG	Gibson Cloning LSM14A-mScarlet (mScarlet fragment)	None
LSM14A_BACK_FWD	GCATGGACGAGCTGTACAAGTACTCAGATCTCGAGCTCAAGCTTCGAATTCCCATG	Gibson Cloning LSM14A-mScarlet (LSM14A backbone)	None
LSM14A_BACK_REV	ACTGCCTCGCCCTTGCTCACCATGGTGGCGACCGGTAGCGCTAGC	Gibson Cloning LSM14A-mScarlet (LSM14A backbone)	None
FRAG_MRB_FWD	CGGGCTGGAGCCACTCGTGCGCCGCCATGCTGTACGCCGCCAACCCTGGGCAGCTCTTC	Generating fragment for RBM TIRR	None

	GGCCGCATCCCCATGCGCTTCTCGGTGCTGATGCAGATGGCCTTCGACGGGCTGCTGGGCTTCCCCGGGGGCGCCGTGGACCGGCGCTTCTGGTCGCTGGAGGACGGCCTGAACCGGGTGCTGGGCCTGGGCCTGGGCTGC		
FRAG_MRB_REV	ACCCGCACGAGGCCCAGCACGGCCAGGCCGTGGTCGCGCGAGTGCACCGCGCTGATCTCCACGGCGTGCAGCTGCTCCAGCGTCAGCTGCCGCGCGTACAGGTGCGCCACGACGCGGTGTGGGCCCTCGGTCAGGTGCGAGCTCAGGTAGTCGGCCTCGGTGAGGCGCAGGCAGCCCAGGCCCAGGCCCAGCACCCGGTT	Generating fragment for RBM TIRR	None
BACK_EGFP_TIRR_MRB_FWD	GTGCTGGGCCTCGTGCGGGTCCCGCTGTACACCCAGAAGGAC	Gibson Cloning LSM14A-mScarlet (GFP backbone)	None
BACK_EGFP_TIRR_MRB_REV	GCACGAGTGGCTCCAGCCCGGCCCTAGGCGCATCGCCTCC	Gibson Cloning LSM14A-mScarlet (GFP backbone)	None
MRB_TIRR_FWD	GGAGGCGATGCGCCTAGGGCCGGGCTGGAGCCACTCGTGC	Gibson Cloning LSM14A-mScarlet (MRB TIRR fragment)	None
MRB_TIRR_REV	CCTTCTGGGTGTACAGCGGGACCCGCACGAGGCCCAGCAC	Gibson Cloning LSM14A-mScarlet (MRB TIRR fragment)	None
TIRR_WT_RED_FWD	AGGTCTATATAAGCAGAGCTCAAGCTTGCGGCCGCCACCATGTCGACGGCGGCGGTTCCGGAGCTGAAGCAGATCAGCCGGGTGGAGGCGATGCGCCTAGGGCCGGGCTGGAGCCACTCGTGCCACGCCATGCTGTACGCCGCCAACCCTGGGCAGCTCTTCGGCCGCATCCCCATGCGC	Generating fragment for green NES mutant (WT segment)	None
TIRR_MUT_RED_FWD	AGGTCTATATAAGCAGAGCTCAAGCTTGCGGCCGCCACCATGT	Generating fragment for red NES mutant (mutant segment)	None

	CGACGGCGGCGGTTCCGGAGGCCAAGCAGGCCAGCCGGGCCGAGGCGGCCCGCGCCGGGCCGGGCTGGAGCCACTCGTGCCACGCCATGCTGTACGCCGCCAACCCTGGGCAGCTCTTCGGCCGCATCCCCATGCGC		
TIRR_WT_GREEN_REV	CAGGTAGTCGGCCTCGGTGAGGCGCAGGCAGCCCAGGCCCAGGCCCAGCACCCGGTTCAGGCCGTCCTCCAGCGACCAGAAGCGCCGGTCCACGAAGCCCCCGGGGAAGCCCAGCAGCCCGTCGAAACGCATCTGCATCAGCACCGAGAAGCGCATGGGGATGCGGCCGAAGAGCTGCCC	Generating fragment for red NES mutant (WT segment)	None
TIRR_MUT_GREEN_REV	CAGGTAGTCGGCCTCGGTGAGGCGCAGGCAGCCGGCGCCGGCGCCGGCGGCCCGGTTGGCGCCGTCCTCGGCCGACCAGAAGCGCCGGTCCACGAAGCCCCCGGGGAAGCCCAGCAGCCCGTCGAAACGCATCTGCATCAGCACCGAGAAGCGCATGGGGATGCGGCCGAAGAGCTGCCC	Generating fragment for green NES mutant (mutant segment)	None
TIRR_WT_BLUE_FWD	CTGAGCTCGCACCTGACCGAGGGCCCACACCGCGTCGTGGCGCACCTGTACGCGCGGCAGCTGACGCTGGAGCAGCTGCACGCCGTGGAGATCAGCGCGGTGCACTCGCGCGACCACGGCCTGGAGGTGCTGGGCCTCGTGCGGGTCCCGCTGTACACCCAGAAGGACCGAGTCGGAGGC	Generating fragment for orange NES mutant (WT segment)	None
TIRR_MUT_BLUE_FWD	CTGAGCTCGCACCTGACCGAGGGCCCACACCGCGCCGCCGCGC	Generating fragment for blue NES mutant (mutant segment)	None

	ACGCCTACGCGCGG CAGGCCACGGCCGA GCAGCTGCACGCCG TGGAGATCAGCGCG GTGCACTCGCGCGA CCACGGCCTGGAGG TGCTGGGCCTCGTGC GGGTCCCGCTGTACA CCCAGAAGGACCGA GTCGGAGGC		
TIRR_WT_ORANGE_REV	GGTGGCTGCAGCCA GGGCCTCAACCAGC TTCTCCTCGGGCATC ATGTTGAGCACCTTG AGGGCAAAGAGGAG CTGGCACTTAGCCGT GCTCACGAAGGCGT TGCTCAGGAAGTTG GGGAAGCCTCCGAC TCGGTCCTTCTGGGT GTACAGCGGGACCC GCACGAGGCCCAGC ACCTCCAG	Generating fragment for blue NES mutant (WT segment)	None
TIRR_MUT_ORANGE_REV	GGTGGCTGCAGCCA GGGCCTCAACCAGC TTCTCCTCGGGGGCG GCGTTGGCCACCTTG GCGGCAAAGGCGGC CTGGCACTTAGCCGT GCTCACGAAGGCGT TGCTCAGGAAGTTG GGGAAGCCTCCGAC TCGGTCCTTCTGGGT GTACAGCGGGACCC GCACGAGGCCCAGC ACCTCCAG	Generating fragment for orange NES mutant (mutant segment)	None
NES_RG_FWD	TGCCTGCGCCTCACC GAGGCC	Gibson Cloning red and green NES mutant	None
NES_RG_REV	TGGTGGCGGCCGCA AGCTTGA	Gibson Cloning red and green NES mutant	None
NES_BO_FWD	AAGCTGGTTGAGGC CCTGGCTGC	Gibson Cloning blue and orange NES mutant	None
NES_BO_REV	GGCCCTCGGTCAGGT GCGAGC	Gibson Cloning red and green NES mutant	None
TIRR_GFP_fwd	ACAGTGGATCCGGA GCTACCATGTCGACG GCGGCGGTTCCGGA G	Gibson Cloning TIRR-GFP Lentivirus Plasmid	None
TIRR_GFP_rev	CTCTAGAGTCGCGGC CTCAGTTACTTGTAC AGCTCGTCCATGCCG AGAGTGATCCC	Gibson Cloning GFP and TIRR-GFP Lentivirus Plasmid	None

Lenti_rev_TIRR_GFP	GGAACCGCCGCCGTCGACATGGTAGCTCCGGATCCACTGTCCACC	Gibson Cloning TIRR-GFP Lentivirus Plasmid	None
Lenti_Backbone_fwd	TGGACGAGCTGTACAAGTAACTGAGGCCGCGACTCTAGAGTCGAC	Gibson Cloning GFP and TIRR-GFP Lentivirus Plasmid	None
GFP_only_fwd	ACAGTGGATCCGGAGCTACCATGGTGAGCAAGGGCGAGGAGCTGT	Gibson Cloning GFP Lentivirus Plasmid	None
Lenti_rev_GFP	TCCTCGCCCTTGCTCACCATGGTAGCTCCGGATCCACTGTCCACC	Gibson Cloning GFP Lentivirus Plasmid	None
FISH_PLA_ZNF600_1	TAGTCACCCTCATCTGAGATGTGCAGACTGCAAGGAAATGAAAAAAAAAAAAAAAAAAAAAAAAAAAAAAAAAAAAAAAATATGACAGAACTAGACAC	FISH-PLA	None
FISH_PLA_ZNF600_2	TTACAACACTTACAAATAAAGTAATTTATTTCACAAATTTAAAAAAAAAAAAAAAAAAAAAAAAAAAAAAAAAAAAAAAATATGACAGAACTAGACAC	FISH-PLA	None
FISH_PLA_ZNF600_3	AACAAACAAACAAAAATTGGAAGTGATGTATCTTCTCACTAAAAAAAAAAAAAAAAAAAAAAAAAAAAAAAAAAAAAAAATATGACAGAACTAGACAC	FISH-PLA	None
FISH_PLA_ZNF600_4	ATCCTTAAAGAATTTTGAAAGTCGACTGTGATCCCAGCTAAAAAAAAAAAAAAAAAAAAAAAAAAAAAAAAAAAAAAAAATATGACAGAACTAGACAC	FISH-PLA	None
FISH_PLA_ZNF600_5	CTGAGGAAGAGCCATGCCTGGCTCCTTTCCTTTCCTCTTAAAAAAAAAAAAAAAAAAAAAAAAAAAAAAAAAAAAAATATGACAGAACTAGACAC	FISH-PLA	None
FISH_PLA_PELI2_1	TTTTTACCCAGCATTAACAATGGTGTAAT	FISH-PLA	None

	CACACACCCTTTCAA GCACATAAAAAAAA AAAAAAAAAAAAAA AAAAAAAAAAAAAA AAAATATGACAGAA CTAGACAC		
FISH_PLA_PELI2_2	AGGCGCATCATAAC CTAAGCCACAAAGG CTAAACTACTGGGA CAGAAGATAAAAAA AAAAAAAAAAAAAA AAAAAAAAAAAAAA AAAAAATATGACAG AACTAGACAC	FISH-PLA	None
FISH_PLA_PELI2_3	AGGGATTCTTCATGA GGGCAAAGACTTAT TTGAAAGGATGTAA GTTATACAAAAAAA AAAAAAAAAAAAAA AAAAAAAAAAAAAA AAAAATATGACAGA ACTAGACAC	FISH-PLA	None
FISH_PLA_PELI2_4	CATCCCCTTAACTTC TAGACTATTTAATAC AGAGAAGCAATCTG TCATCTAAAAAAAA AAAAAAAAAAAAAA AAAAAAAAAAAAAA AAAATATGACAGAA CTAGACAC	FISH-PLA	None
FISH_PLA_PELI2_5	AAGATTTTTTCCTGT AAAATGGCTTGTATT TCCACAGGTACAAT AGTGAAAAAAAAAA AAAAAAAAAAAAAA AAAAAAAAAAAAAA AAAATATGACAGAA CTAGACAC	FISH-PLA	None
FISH_PLA_PELI2_6	ACAGCTGGAGATTA ACTTGGAAGTTAGA GAAGTGAAAAAAAT CAGTTCTAAAAAAA AAAAAAAAAAAAAA AAAAAAAAAAAAAA AAAAAATATGACAG AACTAGACAC	FISH-PLA	None
FISH_PLA_BTUB_1	TGGAAAGGGAAGGA TTCCACTTGACAGAG TGGGACAGACTCCTC CAGAGTAAAAAAAA AAAAAAAAAAAAAA AAAAAAAAAAAAAA	FISH-PLA	None

	AAAATATGACAGAA CTAGACAC		
FISH_PLA_BTUB_2	ACACCTCCCTTGAAG CTGAGATGGGAAAT GGACATACTTAGAA ATTTAGTAAAAAAA AAAAAAAAAAAAAA AAAAAAAAAAAAAA AAAAATATGACAGA ACTAGACAC	FISH-PLA	None
FISH_PLA_BTUB_3	ATGGAAAATCCCAT CCCCTTCCTTATATA GTGACTTCTACCCAC TACCTTAAAAAAAA AAAAAAAAAAAAAA AAAAAAAAAAAAAA AAAATATGACAGAA CTAGACAC	FISH-PLA	None
FISH_PLA_BTUB_4	CTACATGTGTTTTCA GCACCTGGTTGGTTC TAAATGGGATCTGG AGACCCAAAAAAAA AAAAAAAAAAAAAA AAAAAAAAAAAAAA AAAATATGACAGAA CTAGACAC	FISH-PLA	None
FISH_PLA_BTUB_5	AATGGAATGGGCAC CAGAAAGAAATACA GGGTCACCCAGAAT GGCAGAAAAAAAAA AAAAAAAAAAAAAA AAAAAAAAAAAAAA AAAAAATATGACAG AACTAGACAC	FISH-PLA	None
FISH_PLA_SPEN_1	CTGAGCAGCTGTCTT GGTCCGGGAAGGCA GGCCAACAGAGGAA GCGGCGGAAAAAAA AAAAAAAAAAAAAA AAAAAAAAAAAAAA AAAAATATGACAGA ACTAGACAC	FISH-PLA	None
FISH_PLA_SPEN_2	GCGGATGTCGCCGT ACCGGTACTCTTGAA ACAGCTCCCCGCTGG AGTGCAAAAAAAAA AAAAAAAAAAAAAA AAAAAAAAAAAAAA AAAATATGACAGAA CTAGACAC	FISH-PLA	None
FISH_PLA_SPEN_3	AGGCAGCCCCTGGA GTTGGTTACTGGGGG	FISH-PLA	None

	TAACTGTGAGGATA CGGGCCTAAAAAAA AAAAAAAAAAAAAA AAAAAAAAAAAAAA AAAAATATGACAGA ACTAGACAC		
FISH_PLA_SPEN_4	GACAGGGACGGGGG CAGGGGTGGGGGTG GGGGCAGCTTTGGC ATCTGGTGAAAAAA AAAAAAAAAAAAAA AAAAAAAAAAAAAA AAAAAATATGACAG AACTAGACAC	FISH-PLA	None
FISH_PLA_SPEN_5	GGCTGGTGGCACCTT CACCACCCCATCCGC GGCCCTGGGCTGCTC GCTGAAAAAAAAAA AAAAAAAAAAAAAA AAAAAAAAAAAAAA AAATATGACAGAAC TAGACAC	FISH-PLA	None
FISH_PLA_SPEN_6	TGGCACAGTATAGG GGTGCAGTCGGTACT CAGACTGCATGACT AGTACCTAAAAAAA AAAAAAAAAAAAAA AAAAAAAAAAAAAA AAAAATATGACAGA ACTAGACAC	FISH-PLA	None

Supplementary Figure Legends

Supplementary Figure 1

A) Overview of UV RIP-Seq protocol. Made with Biorender.com.

B) TIRR-GFP Flp-IN Trex cells were induced to express TIRR-GFP using Doxycycline at different time points up to 24 hours. A time point of 16 hours was used for RIP-Seq.

C) WB of GFP and TIRR-GFP overexpression in GFP and TIRR-GFP Flp-IN Trex cells. Blots for overexpressed GFP, and overexpressed and endogenous TIRR shown.

D) Analysis of read counts in RIP-seq samples from TIRR-GFP or GFP pull downs from cells with or without Etoposide (ETO) treatment.

E) Heat map analysis showing similarity between RIP-seq samples.

F) Principal Component analysis of top and bottom differentially expressed genes.

G) Principal Component analysis (PCA) of RIP-seq samples.

H) Scree plot analysis of RIP-seq samples.

I) Diagram of FISH-PLA technique for detecting RNA-protein interactions. Made with Biorender.com

J) Left: FISH-PLA of *SPEN* with GFP in a WT TIRR-GFP stable cell line +/- ETO damage and a GFP only stable cell line +ETO. Right: n>30 cells. Significance was determined using Kruskal-Wallis test ($p \leq 0.01$, ****$p \leq 0.0001$).

K) As in J) for *Tubulin*.

Supplementary Figure 2

A) GO analysis of RNAs bound to TIRR in DNA damage conditions.

B) GO analysis of mRNA bound to TIRR in no damage condition.

C) String analysis of mRNA bound to TIRR in damage (ETO) condition.

D) Network presentation of RNAs bound to TIRR in damage conditions, which were identified through GO analysis as significantly enriched in RNAPII transcription regulation.

Supplementary Figure 3

Comparison of features of RNAs bound to TIRR in damage conditions vs the reference genome. Features include: coding sequence length, 5'UTR length, transcript length, 3' UTR length and genome span.

Supplementary Figure 4

A) WB of TIRR knockdown in shGFP and shTIRR HeLa cell lines in -/+ ETO induced DNA damage conditions.

B) Left: IF TIRR knockdown in shGFP and shTIRR HeLa cell lines in + ETO induced DNA damage conditions. Right: n>30 cells. Significance was determined using t-test, (****$p \leq 0.0001$).

C) Heatmap analysis showing similarity between RNA-seq samples from cells expressing shGFP (control) or shTIRR, with or without Etoposide (ETO) treatment.

D) Principal component analysis (PCA) of RNA-seq samples, as in A).

E) Dot plot showing expression levels of TIRR in RNA-seq samples, as in A).

F) Volcano plot showing differentially expressed RNA in samples from cells expressing shTIRR and shGFP in no damage condition.

G) Volcano plot showing differentially expressed RNA in samples from cells expressing shTIRR and shGFP in damage (ETO) condition.

H) Volcano plot showing differentially expressed RNA in samples from cells expressing shGFP in no damage (no ETO) and damage (ETO) condition.

I) Volcano plot showing differentially expressed RNA in samples from cells expressing shTIRR in no damage (no ETO) and damage (ETO) condition.

Supplementary Figure 5

A) Principal component analysis (PCA) of RNA-seq samples from cells expressing shGFP (control) or shTIRR, with or without IR treatment. DNA damage was induced by IR and samples were collected 1 hour later.

B) Dot plot showing expression levels of TIRR in RNA-seq samples, as in A).

C) Volcano plot showing differentially expressed RNA in samples from cells expressing shTIRR and shGFP in no damage (no IR) condition.

D) Volcano plot showing differentially expressed RNA in samples from cells expressing shTIRR and shGFP in damage (IR) condition.

E) Volcano plot showing differentially expressed RNA in samples from cells expressing shGFP in no damage (no IR) and damage (IR) condition.

F) Volcano plot showing differentially expressed RNA in samples from cells expressing shTIRR in no damage (no IR) and damage (IR) condition.

G) Principal component analysis (PCA) of RNA-seq samples from cells expressing shGFP (control) or shTIRR, with or without damage treatment comparing ETO and IR.

Supplementary Figure 6

A) Representative western blot showing protein levels of IRF3, HOXD11, TIRR, γH2AX and Tubulin in cells expressing shGFP and shTIRR with or without Etoposide treatment.

B) Quantification of HOXD11 protein levels, n=3, as in A). Significance was determined using t-test.

C) Quantification of IRF3 protein levels, n=3, as in A). Significance was determined using t-test.

D) Western blot of yH2AX after 2 hrs treatment with etoposide ranging from 0 to 20 µM.

E) Left: Confocal images showing LSM14A foci (P bodies) immunofluorescence (red) and DAPI (blue) when treated with etoposide (0, 5, 10 uM). Right: Quantification of LSM14A foci for 3 images. Significance was determined using t-test.

Supplementary Figure 7

A) Left: Confocal images showing RNA FISH signals (in green) for *ZNF600* in shGFP or shTIRR cells in no damage (-ETO) condition. Right: Quantification of *ZNF600* mRNA signals in nucleus and cytoplasm plotted as relative FISH signal intensity in shGFP or shTIRR cells upon ETO, n>50 cells. Significance was determined using Kruskal-Wallis test, (***$p \leq 0.001$, ****$p \leq 0.0001$).

B) As in A) for *PELI2* mRNA.

C) As in A) for *SPEN* mRNA

D) As in A) for *Tubulin* mRNA

Supplementary Figure 8

A) Left: Confocal images showing RNA FISH signals (in green) for *ZNF600* in shGFP or shTIRR cells in damage (+IR) condition. Right: Quantification of *ZNF600* mRNA signals in nucleus and cytoplasm plotted as relative FISH signal intensity in shGFP or shTIRR cells upon IR, n>50 cells. Significance was determined using Kruskal-Wallis test, (***$p \leq 0.001$, ****$p \leq 0.0001$).

B) As in A) for *PELI2* mRNA.

Supplementary Figure 9

A) Left: Representative confocal images showing immunofluorescence of TIRR in HeLa cells with or without IR treatment and with or without LMB treatment. DAPI was used to stain nuclei. Right: Quantification of TIRR signal in cytoplasm. n>50 cells, significance was determined using Kruskal-Wallis test (****$p \leq 0.0001$).

B) Left: Representative confocal images showing immunofluorescence of TIRR in U2OS cells with or without Etoposide (ETO) treatment and with or without LMB treatment. DAPI was used to stain nuclei. Right: Quantification of TIRR signal in cytoplasm. n>50 cells, significance was determined using Kruskal-Wallis test (****$p \leq 0.0001$).

C) Western blot showing expression levels of TIRR-GFP, TIRR, mCherry-LaminB1-10, LaminB1 and GAPDH in U2OS cells transfected with PA-GFP-TIRR and mCherry-LaminB1-10.

Supplementary Figure 10

A) Left: Confocal images showing PLA of TIRR and XPO1 in cells with or without IR treatment. Single antibody was used as negative control. Right: Quantification of PLA signals, n > 50 cells. Significance was determined using non-parametric Mann-Whitney test (****$p \leq 0.0001$).

B) Western blot of Co-IP of XPO1 with TIRR-GFP in damage and non-damage conditions (Etoposide). A GFP only control was used to assess non-specific binding of XPO1 to the beads.

C) Western blot of Co-IP of TIRR with XPO1-FLAG in damage and non-damage conditions (Etoposide). A mScarlet-FLAG control was used to assess non-specific binding of TIRR to the beads.

D) Western blot of XPO1-FLAG and mScarlet-FLAG constructs compared to a mock transfection control.

E) Western blot of GFP and TIRR-GFP transfected plasmids, including RNA-binding mutant (RBM) and NES mutants (NES1-NES4)

F) Western blot of GFP and TIRR-GFP transfected plasmids, including RNA-binding mutant (RBM), 53BP1-binding mutant (K10E) and NES mutants (NES1-NES4)

G) Left: Confocal images showing PLA of TIRR and XPO1 in cells stably expressing WT-TIRR GFP and transfected with siTIRR, or expressing GFP and transfected with siCTRL or siTIRR. Right: Quantification of PLA signals, n>30 cells. Significance was determined using Kruskal-Wallis test (****$p \leq 0.0001$).

H) Left: Confocal images showing GFP/XPO1 PLA of transfected TIRR-GFP WT, TIRR NES2 and NES4 mutants in cells treated with Etoposide (ETO). Expression of GFP constructs is in green, PLA in red. Quantified cells were selected for similar GFP expression. DAPI indicates nuclei. Bottom: Confocal images showing PLA of TIRR and XPO1 in cells expressing GFP control. Single antibodies were used as negative controls. Right: Quantification of PLA signals, n>30 cells. Significance was determined using non-parametric Kruskal-Wallis test (****$p \leq 0.0001$).

Supplementary Figure 11

A) Representative confocal images showing immunofluorescence of TIRR in cells with or without IR treatment. TIRR expression was visualised in green channel (GFP), P-bodies were labelled using anti-LSM14A antibody and visualised in red channel. White arrows point to P-bodies.

B) Left: proximity ligation assay using anti-TIRR and anti-LSM14A antibodies in no damage (-ETO) or damage (+ETO) conditions in U2OS cells. DAPI was used to label nuclei. PLA signals were visualised in red channel. Single antibodies were used as negative controls. Right: quantification of top. Error bar = mean ±SD, significance was determined usingnon-parametric Mann Whitney test (***$p \leq 0.001$).

C) Left: proximity ligation assay using anti-TIRR and anti-LSM14A antibodies in no damage (-IR) or damage (+IR) conditions in HeLa cells. DAPI was used to label nuclei. PLA signals were visualised in red channel. Single antibodies were used as negative controls. Right: quantification of top. Error bar = mean ±SD, significance was determined using non-parametric Mann Whitney test, (***$p \leq 0.001$).

D) Left: Immunofluorescence analysis showing LSM14A staining in no damage (-IR), and damage (+IR) conditions in cells expressing either shGFP (control) or shRNA targeting TIRR (shTIRR). DAPI was used to stain nuclei. Right: Quantification of TIRR signal in cytoplasm, n>50 cells, significance was determined using t- test, (**$p \leq 0.01$).

E) Left: Immunofluorescence analysis showing LSM14A staining in no damage (-ETO), and damage (+ETO) conditions in cells over-expressing either GFP (control) or TIRR. DAPI was used to stain nuclei. Right: Quantification of TIRR signal in cytoplasm, n>50 cells, significance was determined using t- test, (*$p \leq 0.05$).

F) Left: Immunofluorescence analysis showing LSM14A staining in no damage (-IR), and damage (+IR) conditions in cells over-expressing either GFP (control) or TIRR. DAPI was used to stain nuclei. Right: Quantification of TIRR signal in cytoplasm, n>50 cells, significance

was determined using t- test, (*p $\leq$ 0.05).

Supplementary Figure 12

A) Left: Immunofluorescence analysis showing LSM14A staining in no damage (-ETO), and damage (+ETO) conditions in U2OS cells with or without LMB treatment. DAPI was used to stain nuclei. Right: Quantification of TIRR signal in cytoplasm, n>50 cells, significance was determined using t- test, (**p $\leq$ 0.01, ***p $\leq$ 0.001).

B) Left: Immunofluorescence analysis showing LSM14A staining in no damage (-IR), and damage (+IR) conditions in HeLa cells with or without LMB treatment. DAPI was used to stain nuclei. Right: Quantification of TIRR signal in cytoplasm, n>50 cells, significance was determined using t- test, (*p $\leq$ 0.05, **p $\leq$ 0.01).

Supplementary Figure 13

A) Representative confocal images showing co-localisation of RNA FISH signals (in green) for *SPEN* with LSM14A (in red) in shGFP or shTIRR cells after ETO treatment. n>50 cells. White rectangles mark zoomed in images on the right. White arrows point to P-bodies with FISH signal (in yellow). Quantification of FISH and LSM14A co-localisation signal using Pearson's coefficient, n>50 cells, significance was determined using t- test, (*p $\leq$ 0.05, **p $\leq$ 0.01).

B) As in A) for *Tubulin*.

C) Representative confocal images showing co-localisation of RNA FISH signals (in green) for *ZNF600* with LSM14A (in red) in HeLa cells after IR treatment. n>50 cells. White rectangles mark zoomed in images on the right. White arrows point to P-bodies with FISH signal (in yellow).

D) As in A) for *PELI2* mRNA.

Supplementary Figure 14: TIRR facilitates mRNA export and is associated with P-bodies upon DNA damage

A) Co-IP of 53BP1 with TIRR mutants in ETO-induced damage conditions. U2OS with stably integrated TIRR-GFP WT, TIRR-GFP K10E, TIRR-GFP NES2.

B) WB showing TIRR KD in siTIRR transfected conditions. Blots of 53BP1, XPO1 and LSM14A in CTRL and TIRR KD included.

C) Left: IF of yH2AX in +/- LMB treated conditions in HeLa. Right: Quantification of nuclear mean grey value of yH2AX +/- LMB. Significance was determined using non-parametric Mann-Whitney test, (***$p \leq 0.001$)

D) Immunofluorescence showing γH2AX signal in cells transfected with siTIRR for endogenous TIRR KD, rescued with stably expressing GFP, TIRR-GFP WT, TIRR-GFP K10E, or TIRR-GFP NES2 mutant, at 15 min post 10 Gy IR treatment. Significance was determined using t- test, (**$p \leq 0.01$, ****$p \leq 0.0001$).

E) Immunofluorescence showing γH2AX signal in cells transfected with siTIRR for endogenous TIRR KD, rescued with stably expressing GFP, TIRR-GFP WT, TIRR-GFP K10E, or TIRR-GFP NES2 mutant, at 6 hours post 10 Gy IR treatment. Significance was determined using t- test, (**$p \leq 0.01$, ****$p \leq 0.0001$).
